# Consensus on pre-operative total knee replacement education and prehabilitation recommendations: a UK-based modified Delphi study

**DOI:** 10.1186/s12891-021-04160-5

**Published:** 2021-04-14

**Authors:** Anna M. Anderson, Christine Comer, Toby O. Smith, Benjamin T. Drew, Hemant Pandit, Deborah Antcliff, Anthony C. Redmond, Gretl A. McHugh

**Affiliations:** 1 Leeds Institute of Rheumatic & Musculoskeletal Medicine, University of Leeds, Leeds, UK; 2 NIHR Leeds Biomedical Research Centre, Leeds, UK; 3 Leeds Community Healthcare NHS Trust Musculoskeletal and Rehabilitation Services, Leeds, UK; 4 School of Health Sciences, University of East Anglia, Norwich, UK; 5 Nuffield Department of Orthopaedics, Rheumatology and Musculoskeletal Sciences, University of Oxford, Oxford, UK; 6 School of Healthcare, University of Leeds, Leeds, UK; 7 Physiotherapy Department, Bury Care Organisation, Northern Care Alliance NHS Group, Bury, England, UK

**Keywords:** Total knee replacement, Total knee arthroplasty, Pre-operative care, Education, Prehabilitation, Exercise, Delphi study

## Abstract

**Background:**

Over 90,000 total knee replacement (TKR) procedures are performed annually in the United Kingdom (UK). Patients awaiting TKR face long delays whilst enduring severe pain and functional limitations. Almost 20% of patients who undergo TKR are not satisfied post-operatively. Optimising pre-operative TKR education and prehabilitation could help improve patient outcomes pre- and post-operatively; however, current pre-operative TKR care varies widely. Definitive evidence on the optimal content and delivery of pre-operative TKR care is lacking. This study aimed to develop evidence- and consensus-based recommendations on pre-operative TKR education and prehabilitation.

**Methods:**

A UK-based, three-round, online modified Delphi study was conducted with a 60-member expert panel. All panellists had experience of TKR services as patients (*n* = 30) or professionals (*n* = 30). Round 1 included initial recommendations developed from a mixed methods rapid review. Panellists rated the importance of each item on a five-point Likert scale. Panellists could also suggest additional items in Round 1. Rounds 2 and 3 included all items from Round 1, new items suggested in Round 1 and charts summarising panellists’ importance ratings from the preceding round. Free-text responses were analysed using content analysis. Quantitative data were analysed descriptively. All items rated as ‘Important’ or ‘Very important’ by at least 70% of all respondents in Round 3 were included in the final set of recommendations.

**Results:**

Fifty-five panellists (92%) (patients *n* = 26; professionals *n* = 29) completed Round 3. Eighty-six recommendation items were included in Round 1. Fifteen new items were added in Round 2. Rounds 2 and 3 therefore included 101 items. Seventy-seven of these reached consensus in Round 3. Six items reached consensus amongst patient or professional panellists only in Round 3. The final set of recommendations comprises 34 education topics, 18 education delivery approaches, 10 exercise types, 13 exercise delivery approaches and two other treatments.

**Conclusions:**

This modified Delphi study developed a comprehensive set of recommendations that represent a useful resource for guiding decision-making on the content and delivery of pre-operative TKR education and prehabilitation. The recommendations will need to be interpreted and reviewed periodically in light of emerging evidence.

**Supplementary Information:**

The online version contains supplementary material available at 10.1186/s12891-021-04160-5.

## Background

The demand for total knee replacement (TKR) surgery is rising [1, 2], with over 90,000 TKR procedures already being performed annually in the United Kingdom (UK) [3, 4]. Patients awaiting TKR often experience severe pain, functional limitations and psychological distress [5–7]. Almost 20% of patients are not satisfied following TKR surgery [8, 9]. This is related to multiple factors, of which failure to meet pre-operative expectations is key [8, 10]. Even amongst patients who are satisfied with their TKR, the prevalence of residual symptoms such as swelling, stiffness and functional limitations is high [11–13].

Optimising pre-operative TKR care is an important strategy for addressing the above issues [14–16]. For example, pre-operative TKR education helps set realistic expectations [14, 17] and may reduce pre-operative anxiety [14] and length of hospital stay [18]. Systematic reviews suggest that pre-operative TKR exercise also shortens length of hospital stay and improves post-operative outcomes [16, 19]. Pre-operative exercise is a key component of prehabilitation programmes, which are designed to facilitate patients’ post-operative recovery by optimising their pre-operative health and well-being [20, 21]. A multimodal approach to prehabilitation is however advocated; therefore, prehabilitation programmes may also include other interventions such as smoking cessation and psychological support [20, 21].

Despite the potential benefits of pre-operative TKR education and prehabilitation, current UK pre-operative TKR services vary widely [22–24]. For example, not all hospitals provide a formal pre-operative TKR education and prehabilitation programme [22]. Amongst those that do, there is variation in the programme content and whether it is provided in a group or one-to-one format [22]. A recently published UK National Institute for Health and Care Excellence guideline states that patients awaiting TKR surgery should receive pre-operative information and advice on prehabilitation [22]. This guideline lacks details about the content and delivery of pre-operative TKR care however and, given it identified uncertainties regarding how best to deliver pre-operative information and relied predominantly on low quality underpowered prehabilitation studies, the need for further research in this area is highlighted [22]. This need is pressing because the UK 18-week referral to treatment standard was frequently breached for TKR surgery in 2019 [25] and the COVID-19 (SARS-CoV-2) pandemic is increasing TKR waiting times further [26]. Therefore, patients listed for TKR surgery are currently likely to require prehabilitation for a prolonged period. The COVID-19 pandemic is also catalysing health service redesign [27, 28], presenting an opportune time for research to inform service provision.

The aim of the present study was therefore to develop evidence- and consensus-based recommendations on the content and delivery of pre-operative TKR education and prehabilitation. Previous consensus-based studies have addressed TKR care [29–33]. None of these studies focused exclusively on pre-operative TKR education and prehabilitation however, and their findings cannot be directly applied to the UK context [34]. Additionally, patient representation on their expert panels was minimal or absent [29–33], despite the key role of patients in guideline development [35]. The present study sought to overcome these issues by employing a UK-based expert panel with equal numbers of patient and professional panellists.

The purpose of the recommendations is to help guide clinical practice until more robust evidence on pre-operative TKR education and prehabilitation becomes available. This study is the first phase of a mixed methods project aimed at developing a pre-operative TKR education and prehabilitation digital intervention. Correspondingly, the recommendations will also inform the digital intervention development.

## Methods

**Fig. 1:**
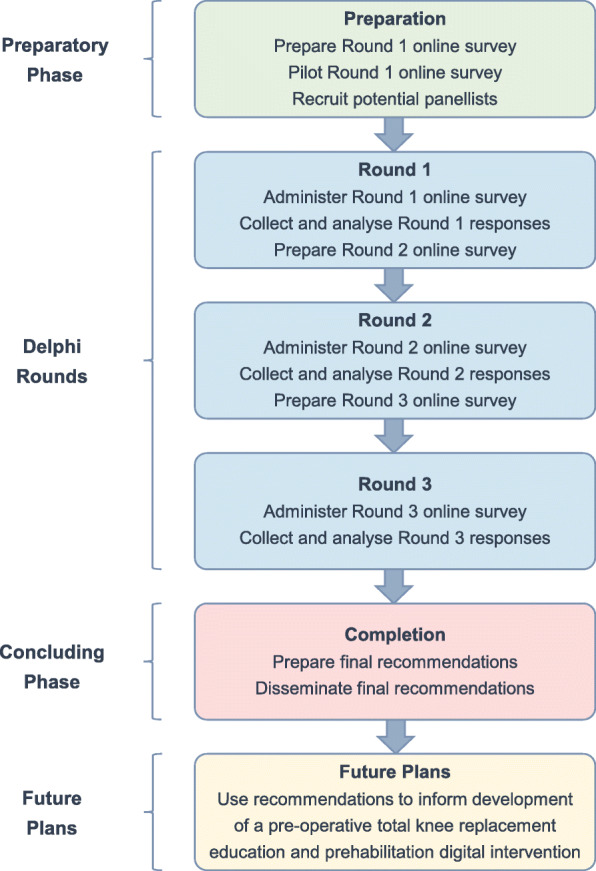
Flow chart of the Delphi process. Flow chart stages based on recommendations from Jünger et al. (2017) [36]

This was a three-round online modified Delphi study (Fig. 1). The study is reported in line with recommendations for the Conducting and REporting of DElphi Studies (CREDES) [36] and proposed Delphi study reporting quality indicators [37]. Ethical approval was obtained from the London – Riverside Research Ethics Committee (Reference number: 19/LO/0813). A Project Advisory Group, involving four project team members, two patient representatives, an independent chair and a key collaborator, oversaw the study. This group met twice during the study, approximately 6 months apart. The first meeting was held prior to data collection. The second meeting was held after completion of data collection.

A modified Delphi technique was employed, in which the initial round was developed from existing evidence [38]. This approach was chosen to minimise the burden on panellists [39, 40] and optimise the quality of the recommendations [41].

### Expert panel

There are no established guidelines on the optimal Delphi study panel size [42]. Therefore, a target of 24 to 70 panellists was set to ensure key stakeholders were sufficiently represented and the panel remained manageable. Panellists were selected using stratified purposive sampling of a minimum number of patients and professionals from key groups, availability sampling of patients and professionals and snowball sampling of professionals [43–45]. It was decided a priori to include a minimum of six patients listed for TKR surgery, six patients who had undergone TKR surgery and two of the following professionals: orthopaedic surgeons, advanced arthroplasty practitioners, nurses, physiotherapists, occupational therapists and clinical commissioners (Additional File 1).

Patients were recruited via social media and Leeds Teaching Hospitals NHS Trust orthopaedic clinics. Although patients were not actively encouraged to share the study details, patients who heard about the study via word-of-mouth were included. Professionals were recruited from all four UK nations via social media, professional networks and encouraging professionals to share the study details with other professionals.

Adults able to communicate in English and use/access the Internet and email were eligible for inclusion if they had experience of TKR services through any of the following:

- Patient who is listed for TKR surgery
- Patient who has undergone TKR surgery within the past 2 years
- Health professional with experience of working with patients undergoing TKR surgery in the NHS
- Clinical commissioner with experience of commissioning orthopaedic services

### Data collection

Data were collected between 13th December 2019 and 19th March 2020. It was decided a priori to include three rounds to increase convergence whilst minimising participant attrition [38, 42, 46] (Fig. 1). All three surveys were hosted using the *Online surveys* tool [47] and administered via email. A consent statement was included on each survey’s introductory page. Panellists were required to complete the consent statement prior to completing the remainder of the survey. Reminders were provided via email and/or telephone to help maximise response rates [38]. All individuals who completed Round 1 were subsequently emailed links to Rounds 2 and 3.

#### Round 1

Round 1 included an initial set of pre-operative TKR education and prehabilitation recommendations (Additional File 2). The initial recommendations were based on a mixed methods rapid review (PROSPERO registration number: CRD42019143248, to be reported in full elsewhere) and covered five sections:

1. Pre-operative TKR education topics (29 items)
2. Pre-operative TKR education delivery (22 items)
3. Pre-operative TKR exercise types (14 items)
4. Pre-operative TKR exercise programme delivery (16 items)
5. Other pre-operative TKR treatments (5 items)

Where appropriate, items included a ‘More info’ option that panellists could click on to read an explanation of that item. Panellists were asked to rate each recommendation item on a five-point Likert scale from ‘Not at all important’ to ‘Very important’. Free-text options were included at the end of each recommendation section to allow panellists to suggest additional items.

Round 1 also included questions on panellists’ characteristics. Separate sets of questions were included for patient panellists (focused on their socio-demographic and clinical characteristics) and professional panellists (focused on their workplace, role and experience).

Prior to full circulation, Round 1 was pilot tested by seven study team members, three physiotherapists, one nurse and three patient representatives. This led to minor wording/structural changes for clarity; amendments to five recommendation items, predominantly to make the items more applicable to clinical practice; inclusion of five new recommendation items, which the pilot testers perceived were important to consider; and inclusion of six additional ‘More info’ options (five of which explained health professional teams’ roles) to assist any panellists who were unfamiliar with the terms used.

No individuals who pilot tested Round 1 joined the main expert panel. Round 1 took panellists a median of 20 min 18 s online time to complete.

#### Round 2

Round 2 included all the recommendation items from Round 1 to ensure all items had equal opportunity of reaching as high a level of consensus as possible [38]. This approach was chosen to enable prioritisation of the items based panellists’ responses in the final round.

**Fig. 2:**
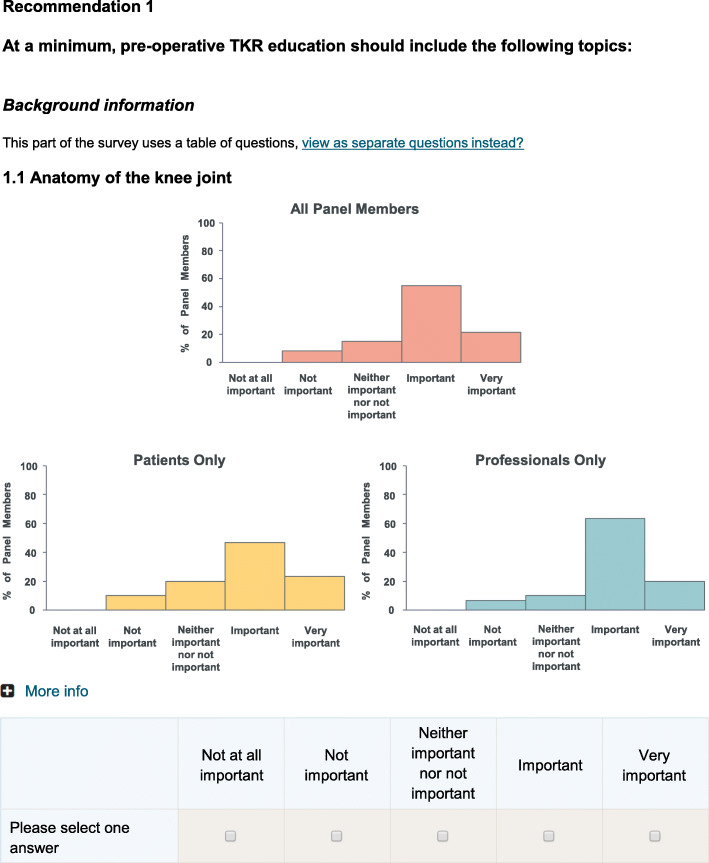
Round 2 recommendation item example

Each item from Round 1 was accompanied by three charts showing panellists’ importance ratings for that item in Round 1 (Fig. 2). Providing panellists with a summary of the results of the preceding round is an established approach for encouraging panellists to reconsider their initial judgement and hence facilitate the development of consensus [42, 48].

Round 2 also included additional recommendation items generated from the Round 1 free-text responses. As in Round 1, panellists were asked to rate the importance of each item using a five-point Likert scale. No free-text options were included in Round 2 to minimise panellist and researcher burden. Round 2 took panellists a median of 17 min 53 s online time to complete.

#### Round 3

Round 3 followed the same format as Round 2, with inclusion of all the Round 2 items accompanied by three charts summarising panellists’ Round 2 importance ratings. As in the preceding rounds, panellists were asked to rate the importance of each item using a five-point Likert scale. Round 3 took panellists a median of 16 min 31 s online time to complete.

### Data analysis

#### Free-text responses

The Round 1 free-text responses were analysed using directed content analysis [49, 50]. This involved creating a ‘formative categorisation matrix’ based on the Round 1 survey (Additional File 3). Each recommendation section was considered a main category. Each recommendation item was considered a potential sub-category. The free-text responses were inductively coded. Where possible, codes were included within the pre-specified sub-categories. Codes that did not fit within the pre-specified sub-categories were included in new, inductively generated sub-categories. The sub-categories were then grouped into generic categories. The generic categories were reviewed to determine whether any new main categories were required. The content analysis was undertaken by one study team member (AMA). All aspects of the analysis were verified by at least one additional study team member (GAM, CC).

All inductively generated sub-categories relating to a pre-operative TKR intervention component or delivery approach were considered potential new items for inclusion in Round 2. It was specified a priori in the protocol that all potential new items would be included, unless that would result in Round 2 taking significantly longer than 30 min to complete, in which case only new items suggested by more than a threshold percentage of panellists would be included. This approach was chosen to help ensure that potentially important items were not omitted from consideration, whilst also ensuring the time burden for panellists remained manageable.

#### Quantitative data

The panellist characteristics and importance ratings were analysed descriptively using Microsoft Excel 2016 and IBM SPSS Statistics 23. There are no established guidelines on how to define consensus in Delphi studies [37], however percent agreement is frequently used [37], and 70% is a commonly specified threshold [51–53]. Consensus was therefore provisionally defined as at least 70% of respondents rating an item as ‘Important’ or ‘Very important’. Specifying a consensus threshold a priori may lead to important items being omitted due to narrowly missing the arbitrary threshold [37]. To account for this, the 70% threshold was reviewed following completion of Round 3 by the Project Advisory Group, who felt no amendments were required.

Responses were analysed for all panellists considered together and for patient and professional panellists separately. All items that reached consensus in Round 3 amongst all respondents considered together were included in the final set of recommendations.

To facilitate use of the recommendations in clinical practice and help guide future research, a prioritised list of recommendations was developed by grouping the recommendation items into three categories:

- Very important recommendations: Items rated as ‘Very important’ by at least 70% of all respondents in Round 3, ranked according to the percentage of ‘Very important’ ratings.
- Important recommendations: Items rated as ‘Important or ‘Very important’ by at least 70% of all respondents in Round 3 (excluding those categorised as ‘Very important’), ranked according to the percentage of ‘Important’ or ‘Very important’ ratings.
- Excluded recommendations: Items rated as ‘Important’ or ‘Very important’ by less than 70% of all respondents in Round 3, ranked according to the percentage of ‘Important’ or ‘Very important’ ratings.

## Results

### Expert panel

**Fig. 3:**
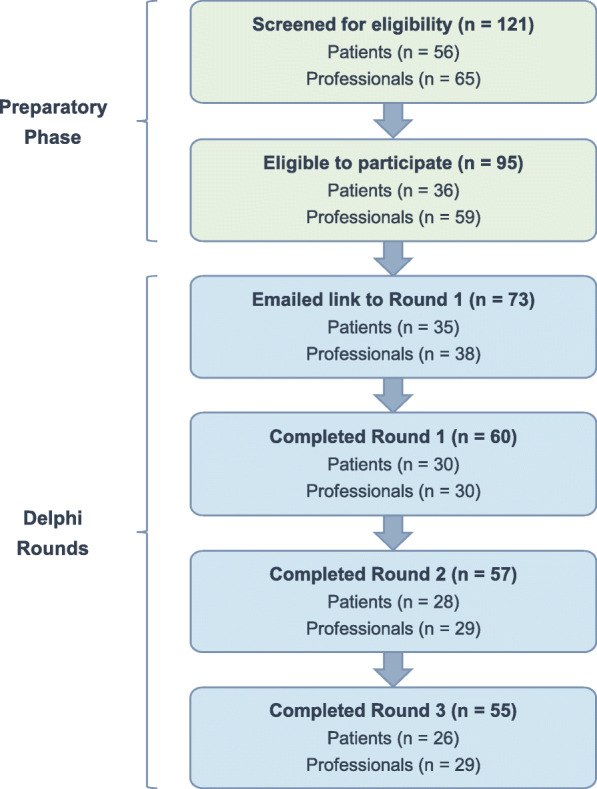
Expert panel flow chart

One hundred and twenty-one individuals were screened, of whom 95 met the eligibility criteria. Twenty-two of these individuals were excluded due to lack of response following the initial contact (one patient, eight professionals) or because a sufficient number of relevant professional panellists had already been recruited (13 professionals). The remaining 73 individuals were emailed the link to Round 1. Sixty individuals (30 patients, 30 professionals) completed Round 1 and formed the expert panel (Fig. 3, reasons for exclusions at each stage available in Additional File 4).

Rounds 2 and 3 were completed by 95 and 92% of panellists respectively. The professional panellist who did not complete Round 2 indicated this was due to being on leave. No other non-respondents provided reasons for non-completion. Tables 1 and 2 provide the patient and professional characteristics respectively.

**Table 1:** Patient panellist characteristics

	Number of panellists (%) (***n*** = 30)
**Living location**	
Scotland	1 (3%)
North East	3 (10%)
North West	5 (17%)
Yorkshire and the Humber	7 (23%)
East Midlands	1 (3%)
West Midlands	3 (10%)
South West	1 (3%)
South East	4 (13%)
East of England	5 (17%)
**Age**	
40–49 years	4 (13%)
50–59 years	9 (30%)
60–69 years	11 (37%)
70–79 years	6 (20%)
**Gender**	
Male	10 (33%)
Female	20 (67%)
**Ethnicity**	
White British	30 (100%)
**Highest educational qualification**	
None	4 (13%)
GCSE / O Level (or equivalent)	5 (17%)
A Level (or equivalent)	2 (7%)
Vocational qualification (or equivalent)	10 (33%)
Undergraduate degree	3 (10%)
Postgraduate degree	6 (20%)
**Current employment status**^**a**^	
Employed full-time	9 (30%)
Employed part-time	7 (23%)
Retired	13 (43%)
Sick leave	2 (7%)
Medically disabled	2 (7%)
**Experience of TKR surgery**^**a**^	
Listed for TKR surgery	11 (37%)
Previously undergone TKR surgery	23 (77%)

*TKR* Total knee replacement

^a^ Panellists could select more than one option

**Table 2:** Professional panellist characteristics

	Number of panellists (%) (***n*** = 30)
**Workplace location**	
Scotland	2 (7%)
Northern Ireland	4 (13%)
Wales	2 (7%)
North West	5 (17%)
Yorkshire and the Humber	9 (30%)
South West	3 (10%)
South East	1 (3%)
London	4 (13%)
**Current professional role**^**a**^	
Orthopaedic surgeon	5 (17%)
Advanced arthroplasty practitioner	2 (7%)
Nurse	2 (7%)
Physiotherapist	12 (40%)
Occupational therapist	4 (13%)
Rehabilitation assistant	3 (10%)
Psychotherapist	1 (3%)
Clinical commissioner	3 (10%)
Manager	2 (7%)
Researcher	2 (7%)
**Years’ experience as a health professional**	
0–9	5 (17%)
10–19	11 (37%)
20–29	9 (30%)
30–49	5 (17%)
**Workplace setting**^**a**^	
NHS teaching hospital	18 (60%)
NHS district/general hospital	7 (23%)
Private Hospital or other private location(s)	5 (17%)
Commissioning organisation	3 (10%)
Increasing Access to Psychological Therapies	1 (3%)
**Currently provide clinical care to patients who are listed for or have undergone TKR surgery**	
Yes	27 (90%)
No	3 (10%)
**Phases of the TKR pathway currently work in (*n*** **= 27)**^**a,b**^	
Pre-operative phase	20 (74%)
Acute phase	20 (74%)
Post-operative phase	19 (70%)
**Number of patients who are listed for or have undergone TKR surgery seen during previous week (*n*** **= 27)**^**b**^	
0	2 (7%)
1–2	4 (15%)
3–5	2 (7%)
6–10	9 (33%)
> 10	10 (37%)

*TKR* Total knee replacement

^a^ Panellists could select more than one option

^b^ Only includes panellists who indicated they currently provide clinical care to patients who are listed for/have undergone total knee replacement surgery

### Free-text responses

Thirty-eight panellists (15 patients, 23 professionals) provided at least one free-text response, resulting in an extensive final categorisation matrix (Additional File 5). The majority of comments fitted within the pre-specified sub-categories. However, 34 new sub-categories were inductively generated. Seven new sub-categories related to TKR care in general, rather than specific intervention components and delivery approaches. These were grouped into a new main category, ‘Planning and prioritising TKR care’ (Additional File 6), and were not included in Round 2.

Across the five pre-specified main categories, 27 new sub-categories were inductively generated. Piloting of Round 2 by study team members suggested that inclusion of all 27 new items could result in the survey taking significantly longer than 30 min to complete. Therefore, in line with protocol, only new items suggested by at least two panellists (3% of all panellists) were included (see Methods, Data analysis, Free-text responses for further details). This resulted in an additional 15 new items being included in Round 2 (Table 3, Additional File 7).

**Table 3:** Recommendation items summary

	Number of recommendation items					
	Round 1		Round 2		Round 3	
	Total^**a**^	Reached consensus^**b**^	Total^**a**^	Reached consensus^**b**^	Total^**a**^	Reached consensus^**b**^
Pre-operative TKR education topics	29	28	35	34	35	34
Pre-operative TKR education delivery	22	14	25	17	25	18
Pre-operative TKR exercise types	14	7	17	8	17	10
Pre-operative TKR exercise delivery	16	8	19	11	19	13
Other pre-operative TKR treatments	5	0	5	1	5	2
**All recommendation items**	**86**	**57**	**101**	**71**	**101**	**77**

*TKR* Total knee replacement

^a^ Total number of recommendation items included in the round

^b^ Number of recommendation items in the round that reached consensus. Consensus was defined as at least 70% of respondents rating an item as ‘Important’ or ‘Very important’

### Importance ratings overview

Based on the rapid review, eighty-six recommendation items were included in Round 1. Fifteen new items were added in Round 2. Seventy-seven of the resulting 101 items were included in the final set of recommendations (Tables 3, 4, 5, 6, 7, 8, Additional File 8). The importance ratings of patient and professional panellists were largely similar (Additional File 9). Overall however, patient panellists provided lower importance ratings than professional panellists, especially during Round 1. The number of items that reached consensus amongst patient but not professional panellists was three, five and one in Rounds 1, 2 and 3 respectively. The number of items that reached consensus amongst professional but not patient panellists was 13, six and five in Rounds 1, 2 and 3 respectively. Sixteen recommendation items were prioritised as ‘Very important’ and 61 were prioritised as ‘Important’ (Additional File 10).

**Table 4:** Pre-operative total knee replacement education topics: Importance ratings summary

Pre-operative total knee replacement education topic item^**a**^	% Important or Very Important rating		
	Round 1 (***n*** = 60)	Round 2 (***n*** = 57)	Round 3 (***n*** = 55)
1.1 Anatomy of the knee joint	77	91	95
1.2 Health conditions that may contribute to needing TKR surgery	77	95	91
1.3 Alternative treatment options to TKR surgery	82	91	87
**1.4 Purpose of pre-operative rehabilitation**	98	98	98
**1.5 Patient involvement in their own management**	98	100	98
1.6 Goal setting	88	93	96
1.7 Using heat and cold	87	88	85
1.8 Obtaining and using walking aids and other equipment	95	96	95
**1.9 Making home preparations**	98	98	100
**1.10 Arranging social support**	88	95	96
1.11 Arranging transport to and from the hospital	82	91	98
**1.12 What to expect during the hospital stay**	98	100	100
1.13 What a TKR surgical procedure involves	92	89	93
**1.14 Risks of TKR surgery and how to minimise them**	97	100	100
**1.15 Common issues that may occur following TKR surgery which do not need to cause alarm**	93	98	100
**1.16 Pain expectations**	97	100	100
**1.17 What to expect following discharge**	95	98	100
**1.18 Recovery expectations**	98	98	98
**1.19 Pain management**	100	100	100
**1.20 Rehabilitation following TKR surgery**	100	100	100
*1.21 Complementary and alternative therapies*	28	21	27
1.22 Returning to daily activities	93	100	98
1.23 Returning to driving and other types of travel	95	96	98
1.24 Returning to sports and leisure activities	90	89	96
1.25 Returning to work	88	95	95
1.26 Physical activity	95	98	100
1.27 Weight management	90	98	100
1.28 Stopping smoking	80	84	85
1.29 Avoiding alcohol misuse	73	82	87
1.30 Optimising management of diabetes	N/A	77	82
1.31 Education for other people, such as carers	N/A	82	91
1.32 Swelling	N/A	98	100
1.33 Organising help if complications occur	N/A	100	100
1.34 Returning to a normal walking pattern	N/A	93	98
1.35 Emotional well-being	N/A	89	93

*N/A* Not applicable because the item was not included in Round 1

*TKR* Total knee replacement

^a^ Consensus was defined as at least 70% of respondents rating an item as ‘Important’ or ‘Very important’. The item in italics did not reach consensus in Round 3 and hence was excluded from the final list of recommendations. Items in bold were prioritised as ‘Very important’. Items in plain text were prioritised as ‘Important’

**Table 5:** Pre-operative total knee replacement education delivery: Importance ratings summary

Pre-operative total knee replacement education delivery item^**a**^	% Important or Very Important rating		
	Round 1 (***n*** = 60)	Round 2 (***n*** = 57)	Round 3 (***n*** = 55)
2.1 Informed by a multidisciplinary team	75	91	96
2.2.1 Informed by members of the orthopaedic surgery team	82	93	95
2.2.2 Informed by members of the nursing team	72	86	95
**2.2.3 Informed by members of the physiotherapy team**	95	98	100
2.2.4 Informed by members of the occupational therapy team	80	88	85
*2.2.5 Informed by members of the social work team*	42	42	31
2.3 Informed by patients who have previously had TKR surgery	63	67	73
2.4 Provide examples of other patients’ experiences of TKR surgery	70	77	76
2.5.1 Delivered using face-to-face group sessions	78	89	89
*2.5.2 Delivered using face-to-face individual sessions*	45	56	56
2.5.3 Delivered using a booklet or other written format	88	98	100
*2.5.4 Delivered using a video or DVD*	63	65	64
2.5.5 Delivered using a website or other electronic format	72	74	75
*2.5.6 Delivered using telephone*	25	18	18
*2.5.7 Delivered using a PowerPoint presentation*	32	23	25
2.6 Delivered using a combination of more than one format	87	93	93
2.7 Delivered through a combination of information provision and an opportunity to actively take part in tasks	78	95	96
**2.8 Provide an opportunity for questions to be addressed**	97	100	100
2.9 Provide an opportunity for a family member/friend to be involved	80	91	93
2.10 Tailored to each patient’s needs	85	82	91
*2.11 Tailored to the right or left knee*	13	7	2
2.12 Received separately from patients waiting for other types of surgery	67	75	76
2.13 Delivered within 4 weeks of TKR surgery	N/A	79	84
*2.14 Delivered in a hospital setting*	N/A	33	22
2.15 Standardised across the UK	N/A	72	80

*N/A* Not applicable because the item was not included in Round 1

*TKR* Total knee replacement

^a^ Consensus was defined as at least 70% of respondents rating an item as ‘Important’ or ‘Very important’. Items in italics did not reach consensus in Round 3 and hence were excluded from the final list of recommendations. Items in bold were prioritised as ‘Very important’. Items in plain text were prioritised as ‘Important’

Additional Files 2 and 7 provide the exact wording of each item

**Table 6:** Pre-operative total knee replacement exercise types: Importance ratings summary

Pre-operative total knee replacement exercise type item^**a**^	% Important or Very Important rating		
	Round 1 (***n*** = 60)	Round 2 (***n*** = 57)	Round 3 (***n*** = 55)
**3.1 Leg strengthening exercises**	98	100	100
*3.2 Arm strengthening exercises*	43	51	64
**3.3 Leg flexibility exercises**	83	95	98
*3.4 Arm flexibility exercises*	27	25	29
*3.5 Torso flexibility exercises*	43	32	27
3.6 Balance exercises	85	95	100
3.7 Functional movement exercises	87	96	95
3.8 Functional technique exercises	80	89	91
*3.9 Warm-up exercises*	58	60	55
*3.10 Cool-down exercises*	48	44	36
3.11 Cardiovascular exercises	60	67	75
3.12 Core control exercises	60	68	76
3.13 Walking practice with walking aids	83	88	91
3.14 Training on steps	83	91	95
3.15 Practicing post-operative exercises	N/A	89	96
*3.16 Water-based exercises*	N/A	32	25
*3.17 Exercises in which the foot does not move*	N/A	39	18

*N/A* Not applicable because the item was not included in Round 1

^a^ Consensus was defined as at least 70% of respondents rating an item as ‘Important’ or ‘Very important’. Items in italics did not reach consensus in Round 3 and hence were excluded from the final list of recommendations. Items in bold were prioritised as ‘Very important’. Items in plain text were prioritised as ‘Important’

**Table 7:** Pre-operative total knee replacement exercise programme delivery: Importance ratings summary

Pre-operative total knee replacement exercise delivery item^**a**^	% Important or Very Important rating		
	Round 1 (***n*** = 60)	Round 2 (***n*** = 57)	Round 3 (***n*** = 55)
*4.1.1 Delivered using an individual instruction session*	52	53	44
4.1.2 Delivered using supervised exercise sessions	73	79	89
4.1.3 Delivered using unsupervised exercise sessions	58	72	91
*4.1.4 Delivered using telephone-delivered sessions*	5	9	5
4.1.5 Delivered using a booklet or other written format	87	93	93
4.2 Delivered using a combination of more than one format	87	96	91
*4.3.1 Take place in the patient’s own home*	53	46	36
*4.3.2 Take place in a clinical setting*	52	47	47
*4.3.3 Take place in a community setting*	52	40	33
*4.4.1 Include high intensity exercises*	33	14	22
4.4.2 Include low to moderate intensity exercises	75	91	98
4.5 Tailored to the patient’s ability	93	96	96
4.6 Be progressive	82	91	87
4.7 Each session should last a minimum of 15 min	63	63	80
4.8 Involve a minimum of 2 sessions per week	78	79	84
4.9 Ideally be performed for a minimum of 6 weeks	80	88	89
4.10 Tailored to each patient’s needs	N/A	88	93
4.11 Provide an opportunity for peer support	N/A	65	75
4.12 Include goal setting	N/A	79	87

*N/A* Not applicable because the item was not included in Round 1

^a^ Consensus was defined as at least 70% of respondents rating an item as ‘Important’ or ‘Very important’. Items in italics did not reach consensus in Round 3 and hence were excluded from the final list of recommendations. Items in plain text were prioritised as ‘Important’

Additional Files 2 and 7 provide the exact wording of each item

**Table 8:** Other pre-operative total knee replacement treatments: Importance ratings summary

Other pre-operative total knee replacement treatment item^**a**^	% Important or Very Important rating		
	Round 1 (***n*** = 60)	Round 2 (***n*** = 57)	Round 3 (***n*** = 55)
5.1 Patients who have a BMI of 27 kg/m^2^ or over should be referred to a weight management programme	67	67	73
5.2 Patients who have been formally diagnosed anxiety or depression should be offered CBT-based therapy	67	74	78
*5.3 Patients should be offered motivational interviewing*	38	37	33
*5.4 Patients should be offered neuromuscular electrical stimulation*	17	5	4
*5.5 Patients should be offered electro-acupuncture*	8	0	0

*BMI* Body Mass Index

*CBT* Cognitive behavioural therapy

^a^ Consensus was defined as at least 70% of respondents rating an item as ‘Important’ or ‘Very important’. Items in italics did not reach consensus in Round 3 and hence were excluded from the final list of recommendations. Items in plain text were prioritised as ‘Important’

Additional File 2 provides the exact wording of each item

### Pre-operative TKR education topics

Twenty-nine education topic items were included in Round 1. Six were added in Round 2. Thirty-four of the resulting 35 items were included in the final set of recommendations (Table 4). All 34 included items reached consensus amongst patient and professional panellists in Round 3 (Additional File 9). Twelve of the included education topic items were prioritised as ‘Very important’ and 22 were prioritised as ‘Important’ (Table 4, Additional File 10).

### Pre-operative TKR education delivery

Twenty-two education delivery items were included in Round 1. Three were added in Round 2. Eighteen of the resulting 25 items were included in the final set of recommendations (Table 5). All 18 included items reached consensus amongst patient and professional panellists in Round 3 (Additional File 9). Informed by members of the physiotherapy team (Item 2.2.3) and provide an opportunity for questions to be addressed (Item 2.8) were prioritised as ‘Very important’. The remaining 16 included education delivery items were prioritised as ‘Important’ (Table 5, Additional File 10).

### Pre-operative TKR exercise types

Fourteen exercise type items were included in Round 1. Three were added in Round 2. Ten of the resulting 17 items were included in the final set of recommendations (Table 6). Cardiovascular exercises (Item 3.11) and core control exercises (Item 3.12) reached consensus amongst professional but not patient panellists in Round 3 (Additional File 9). Additionally, arm strengthening exercises (Item 3.2) reached consensus amongst patient but not professional panellists in Round 3. Arm strengthening exercises did not reach consensus amongst all panellists considered together and therefore was excluded from the final recommendations. Leg strengthening exercises (Item 3.1) and leg flexibility exercises (Item 3.3) were prioritised as ‘Very important’. The remaining eight included exercise type items were prioritised as ‘Important’ (Table 6, Additional File 10).

### Pre-operative TKR exercise programme delivery

Sixteen exercise programme delivery items were included in Round 1. Three were added in Round 2. Thirteen of the resulting 19 items were included in the final set of recommendations (Table 7). An opportunity for peer support (Item 4.11) reached consensus amongst professional but not patient panellists in Round 3 (Additional File 9). All 13 included exercise programme delivery items were prioritised as ‘Important’ (Table 7, Additional File 10).

### Other pre-operative TKR treatments

Five other pre-operative TKR treatment items were included in Round 1 and none were added in Round 2. Referral of patients with a body mass index (BMI) of 27 kg/m^2^ or over to a weight management programme (Item 5.1) and offering cognitive behavioural (CBT)-based therapy to patients who have been formally diagnosed with anxiety or depression (Item 5.2) were included in the final recommendations (Table 8). Both of these items reached consensus amongst professional but not patient panellists in Round 3 (Additional File 9) and were prioritised as ‘Important’ (Table 8, Additional File 10).

## Discussion

This UK-based modified Delphi study developed a comprehensive set of recommendations on the content and delivery of pre-operative TKR education and prehabilitation. Of the 77 items included in the final recommendations, the largest proportion are education topics (Tables 3-4). Smaller proportions are education delivery approaches, exercise delivery approaches and exercise types (Tables 5, 6, 7). A minority of the items are other treatments not focused on education or exercise (Table 8). Correspondingly, the level of agreement between panellists was greater for the education topics section than for any other section, with 12 education topics receiving ‘Important’ or ‘Very important’ ratings from 100% of panellists in the final round (Table 4).

The importance ratings of patient and professional panellists were broadly similar. However, arm strengthening exercises reached consensus amongst patient but not professional panellists only in the final round (Item 3.2; Additional File 9). Conversely, cardiovascular exercises, core control exercises, an opportunity for peer support, referral to a weight management programme and CBT-based therapy all reached consensus amongst professional but not patient panellists in the final round (Items 3.11, 3.12, 4.11, 5.1, 5.2; Additional File 9). Reasons for this could not be explored in the current study. Qualitative research suggests some patients awaiting TKR believe ‘A mechanical problem requires a mechanical fix’, impairing their engagement with nonsurgical interventions [54]. This may partly explain some of the lower importance ratings amongst patient panellists compared to professional panellists.

The large number of education topics included in the final recommendations corresponds with qualitative literature highlighting the importance of comprehensive pre-operative TKR education [15, 17]. This study’s findings also support and expand those of previous Delphi studies [29–33]. The most relevant previous study is that of Westby et al. (2018), a Canadian study that lists quality indicators on pre-operative TKR education, exercise and weight management [29]. These largely align with the findings of the present study, although this study adds detail and there are disparities in the exercise types advocated. For example, pre-operative balance exercises are not specifically mentioned by Westby et al. (2018) [29], but were rated as ‘Important’ or ‘Very important’ by 100% of panellists in final round of this study (Item 3.6; Table 6). Additionally, pre-operative arm strengthening exercises are listed by Westby et al. (2018) [29], but were excluded from the present study’s final recommendations (Item 3.2; Table 6). These disparities might be related to the differing methodology, expert panel composition and healthcare context of this study compared to the study of Westby et al. (2018) [29, 34, 42]. The disparities might also reflect uncertainties in the current pre-operative TKR exercise evidence base [16].

### Strengths and limitations

A key strength of this study is the rigorous application of a modified Delphi technique. Round 1 was developed from existing evidence to optimise the quality of the recommendations [41]. The web-based interface ensured that anonymity between panellists was maintained, which minimises social pressures and avoids group decisions being dominated by specific individuals [36]. Remote data collection facilitated inclusion of geographically dispersed panellists, with all four UK nations being represented (Tables 1-2). Another strength is the broad range of patients and professionals involved in the expert panel, with 87 and 97% of patient and professional panellists completing the final round respectively. This is likely to increase acceptance of the recommendations [42, 55].

This study also presents limitations. Notably, inclusion of all the items in the final recommendations was determined solely by expert consensus rather than empirical data. Therefore, the recommendations need to be interpreted and reviewed in light of emerging evidence. Free-text responses were only included in Round 1 and panellists were not asked to prioritise items, preventing an in-depth exploration of aspects such as the optimal exercise programme duration. Providing panellists with their individual responses from the preceding round may assist their decision-making [55], but this approach was not employed, primarily due to the restricted functionality of the *Online surveys* tool. All panellists were required to be able to use/access the Internet and email and the patient panellists were not necessarily fully representative of all patients undergoing TKR surgery (Table 1). In particular, 100% of the patient panellists identified as White British. Contributory factors to this may have included the requirement of panellists to be able to communicate in English and racial disparities in rates of TKR surgery [56].

### Implications for practice and future research

Definitive evidence on pre-operative TKR education and prehabilitation is currently lacking [16, 18]. Therefore, at present, the recommendations developed in this study provide a useful resource for helping to guide UK health professionals’ decision-making on pre-operative TKR service provision. This could improve patient outcomes by reducing unwarranted variations between services and enhancing care quality. The large number of items included in the final recommendations may be off-putting to clinical decision-makers. The prioritised list of recommendations (Additional File 10) could however be used to select a limited number of recommendations that are relevant locally. The prioritised recommendations also provide a valuable resource for guiding future research on pre-operative TKR interventions.

Arguably, the most challenging and costly recommendations to implement in clinical practice will be referral of patients with a BMI of 27 kg/m^2^ or over to a weight management programme and referral of patients with anxiety or depression to CBT-based therapy if not already provided (Items 5.1, 5.2; Table 8). Neither of these recommendations are currently addressed in standard UK TKR pathways. Two panellists commented the BMI threshold of 27 kg/m^2^ is quite low. Future research investigating whether specific subgroups of patients benefit from pre-operative TKR weight management and psychological support is therefore warranted.

Use of the recommendations is likely to be affected by the COVID-19 pandemic. The recommended education and exercise delivery approaches need to be interpreted with consideration of the new impetus for remote models of care [28, 57]. Digital interventions offer a particularly valuable approach for delivering TKR care remotely at relatively low cost [58–60], making this an important area for future research. The present study’s authors intend to help address this by using the final set of recommendations to inform a future pre-operative TKR education and prehabilitation digital intervention. The COVID-19 pandemic is also substantially increasing the length of time patients remain on the waiting list for TKR surgery [26]. Correspondingly, another key consideration is how to support patients to engage with a pre-operative TKR exercise programme for a prolonged period. Research addressing this, and the disparities regarding pre-operative TKR exercise types noted above, would therefore be valuable.

## Conclusions

This UK-based modified Delphi study developed a comprehensive set of recommendations on pre-operative TKR education and prehabilitation. These cover 34 education topics, 18 education delivery approaches, 10 exercise types, 13 exercise delivery approaches and two other pre-operative treatments. Due to the absence of definitive evidence in this area, inclusion of items in the final recommendations was based solely on expert consensus. Therefore, these recommendations will need to be interpreted and reviewed as necessary in light of new evidence. Until such evidence emerges, the recommendations provide a useful resource for helping to guide health professionals’ decision-making on pre-operative TKR service provision.

## Supplementary Information

**Additional file 1:** Sampling strategy. Sampling strategy used to select panellists for inclusion in the study (**Supplementary Table 1**).

**Additional file 2.** Round 1 survey. Round 1 survey administered during the study.

**Additional file 3: **Formative categorisation matrix. Formative categorisation matrix developed from the Round 1 survey (**Supplementary Table 2**).

**Additional file 4: **Recruitment flow charts. Patient recruitment flow chart (**Supplementary Fig. 1**) and professional recruitment flow chart (**Supplementary Fig. 2**).

**Additional file 5: **Final categorisation matrix. Final categorisation matrix developed from the content analysis of the Round 1 free-text responses (**Supplementary Table 3**).

**Additional file 6: **Inductively generated main category. Inductively generated main category developed during the content analysis of panellists’ Round 1 free-text responses (**Supplementary Table 4**).

**Additional file 7: **Round 2 new items. New items generated from panellists’ Round 1 free-text responses and included in the Round 2 survey (**Supplementary Table 5**).

**Additional file 8.** Final list of recommendations. Final list of recommendations developed from the Round 3 results.

**Additional file 9: **Detailed importance ratings results. Detailed importance rating results for Round 1 (**Supplementary Tables 6–10**), Round 2 (**Supplementary Tables 11–15**) and Round 3 (**Supplementary Tables 16–20**).

**Additional file 10: **Prioritised list of recommendations. Prioritised list of recommendations developed from the Round 3 results (**Supplementary Tables 21–25**).

## Data Availability

The datasets used and/or analysed during the current study are available from the corresponding author on reasonable request.

## Abbreviations

BMI: Body mass index
CBT: Cognitive behavioural therapy
NHS: National health service
TKR: Total knee replacement
UK: United Kingdom
