# Supplementary material for: Consensus on pre-operative total knee replacement education and prehabilitation recommendations: a UK-based modified Delphi study
Source: BMC Musculoskelet Disord. 2021 Apr 14;22:352. doi: 10.1186/s12891-021-04160-5 (PMC8044503; doi:10.1186/s12891-021-04160-5)
Supplement: Supplementary file 1 — Additional file 1: Sampling strategy. Sampling strategy used to select panellists for inclusion in the study (Supplementary Table 1). [file 12891_2021_4160_MOESM1_ESM.docx]

**Consensus on pre-operative total knee replacement education and prehabilitation recommendations: A UK-based modified Delphi study**

**Additional File 1: Sampling strategy**

**Supplementary Table 1: Sampling strategy**

| **Panellist group** | **Experience of TKR surgery** | **Number of panellists** | **Minimum group total** | **Maximum group total** |
| --- | --- | --- | --- | --- |
| Patient | Patient who is listed for TKR surgery | ≥ 6 | 12 | 35 |
|  | Patient who has undergone TKR surgery | ≥ 6 |  |  |
| Professional | Orthopaedic surgeon | ≥ 2 | 12 | 35 |
|  | Advanced arthroplasty practitioner | ≥ 2 |  |  |
|  | Nurse | ≥ 2 |  |  |
|  | Physiotherapist | ≥ 2 |  |  |
|  | Occupational therapist | ≥ 2 |  |  |
|  | Clinical commissioner | ≥ 2 |  |  |
|  | | **Overall total** | 24 | 70 |

*TKR* total knee replacement

Stratified purposive sampling was used to select a minimum number of individuals from the groups specified. Professionals from groups not specified were also eligible for inclusion as long as they met the study eligibility criteria.
