# Supplementary material for: Consensus on pre-operative total knee replacement education and prehabilitation recommendations: a UK-based modified Delphi study
Source: BMC Musculoskelet Disord. 2021 Apr 14;22:352. doi: 10.1186/s12891-021-04160-5 (PMC8044503; doi:10.1186/s12891-021-04160-5)
Supplement: Supplementary file 2 — Additional file 2. Round 1 survey. Round 1 survey administered during the study. [file 12891_2021_4160_MOESM2_ESM.docx]

**Consensus on pre-operative total knee replacement education and prehabilitation recommendations: A UK-based modified Delphi study**

**Additional File 2: Round 1 survey**

*The following survey was hosted on Online Surveys and administered via email. Panellists were not able to proceed with completing the survey until they had completed the Consent Statement.*

*Apart from for the Consent Statement questions, panellists were not able to see the question numbers, but were able to see the page numbers and titles. Unless otherwise specified, all questions were required.*

*Where indicated, items included ‘More info’ options that panellists could click on to read an explanation of the item. An example of a ‘More info’ option is provided below:*

*Recommendation 2.1*

*Pre-operative TKR education should be informed by a multi-disciplinary team rather than members of a single profession*

*More info*

*A multidisciplinary team involves a group of healthcare professionals from different backgrounds, such as surgeons, nurses and physiotherapists, working together.*

*Panellists were asked to rate the importance of each recommendation item on the following scale:*


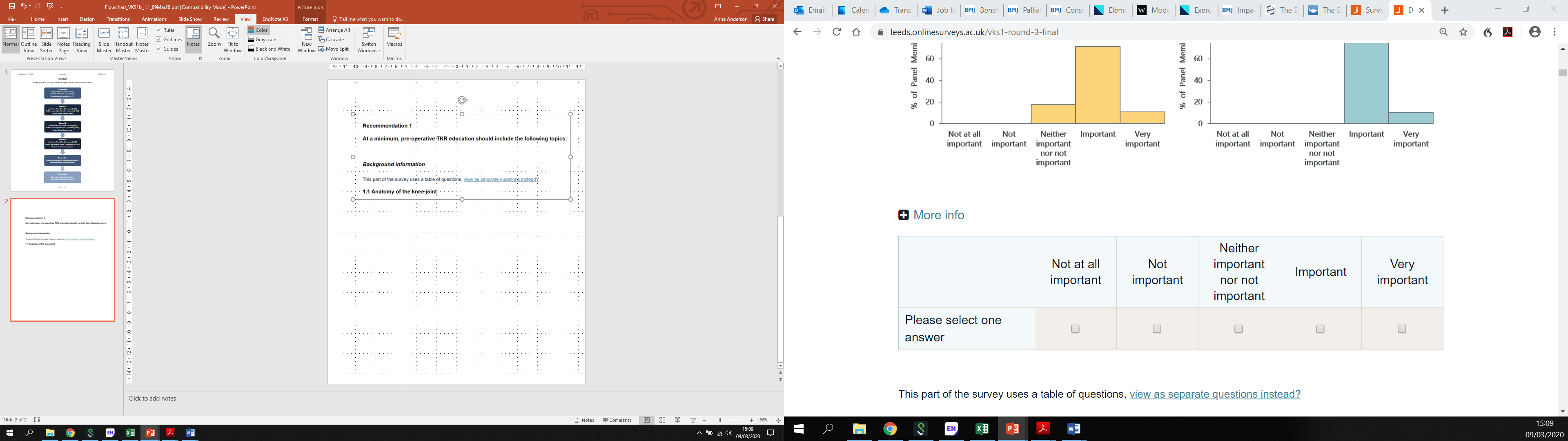


# Page 1: Introduction

Thank you very much for offering to take part in this research study.

The aim of the study is to develop recommendations on pre-operative care for patients waiting for total knee replacement (TKR) surgery. Taking part will involve completing three online questionnaires over a period of approximately three months. This is the first questionnaire in the study.

Further details about this study are provided in the Participant Information Sheet (version 2.0, dated 08/10/2019). You have already received this Participant Information Sheet. However, you can also access it by clicking here.

This questionnaire includes a section on demographic details, such as your experience of TKR surgery, and the following five sections on pre-operative TKR care:

1. Pre-operative education topics
2. Pre-operative education delivery
3. Pre-operative exercise types
4. Pre-operative exercise programme delivery
5. Other pre-operative treatments

The five sections on pre-operative TKR care include an initial list of recommendations on pre-operative care for patients waiting for TKR surgery. These recommendations have been developed based on a review of previous research studies and feedback from patients and professionals. However, there is not good quality evidence to support most of these initial recommendations. We would therefore like you to rate how important you think each recommendation is so that we can develop a finalised list of recommendations.

You will be asked to rate the importance of each recommendation on a scale which includes the following options:

- Not at all important
- Not important
- Neither important nor not important
- Important
- Very important

Please only rate a recommendation as ‘Important’ or ‘Very important’ if you think it should be included in the final list of recommendations.

You will also have the opportunity to suggest additional recommendations if you would like to.

If you have any questions about this study, please contact one of the following people:

Anna Anderson, NIHR Doctoral Fellow in Physiotherapy, [anna.anderson6@nhs.net](mailto:anna.anderson6@nhs.net), 0113 3924920

Professor Anthony Redmond, Professor of Clinical Biomechanics and Head of Section of Clinical Biomechanics and Physical Medicine, [A.Redmond@leeds.ac.uk](mailto:A.Redmond@leeds.ac.uk), 0113 3924914

**Consent Statement (version 2.1, 28/11/19)**

If you are happy to take part in this study, please select ‘Yes’ for each of the following seven statements.

1. I confirm that I have read and understand the Participant Information Sheet (version 2.0; dated 08/10/2019) for this study and have had the opportunity to ask questions.

- Yes

1. I agree to take part in this study, which will involve completing three online questionnaires.

- Yes

1. I understand that my participation in this study is voluntary and I am free to withdraw at any time.

- Yes

1. I understand that, even if I withdraw from this study, the information I have already provided will be used in analysing the results of this study.

- Yes

1. I agree to direct quotes from my responses to the first questionnaire being used in the second and third questionnaires, and in reports, publications and presentations. I understand that these quotes will be anonymised.

- Yes

1. I understand that the information collected during this study may be used to support other research in the future and may be shared anonymously with other researchers.

- Yes

1. I agree to my personal information being stored for the purposes of this study. I understand that any information which could identify me will be kept strictly confidential.

- Yes

1. Please enter your name in the box below.
2. Please enter today’s date in the box below.

# Page 2: Demographic questions

This section is about demographic details, such as your experience of total knee replacement (TKR) surgery. It is important for us to collect this information so that we know whether the people who take part in this study have similar characteristics to the people the recommendations apply to.

Please answer all the following questions.

1. Do you have experience of TKR surgery as a patient or professional?

- Patient
- Professional

*Panellists only saw questions 10a – 10g if they selected ‘Patient’ for question 10*

10a. What region of the UK do you currently live in?

- Scotland
- Northern Ireland
- Wales
- North East
- North West
- Yorkshire and the Humber
- East Midlands
- West Midlands
- South West
- South East
- East of England
- London

10b. What is your current age category?

- Less than 40 years old
- 40 – 49 years old
- 50 – 59 years old
- 60 – 69 years old
- 70 – 79 years old
- 80 years old or over

10c. What is your gender?

- Male
- Female
- Other (if selected, were asked to specify)

10d. What is your ethnic group?

- White British
- White Irish
- White Other
- Indian
- Pakistani
- Bangladeshi
- Chinese
- Black African
- Black Caribbean
- Black Other
- Arab
- Mixed / Multiple Ethnic Background
- Other (if selected, were asked to specify)

10e. What is the highest educational qualification you have completed?

- None
- GCSE / O Level (or equivalent)
- A Level (or equivalent)
- Vocational qualification
- Undergraduate degree
- Postgraduate degree

10f. What is your current employment status? (Please select all options that apply)

- Employed full-time
- Employed part-time
- Self-employed
- Retired
- Unemployed
- Sick leave
- Medically disabled
- Home maker
- In education

10g. What is your experience linked to TKR surgery? (Please select all options that apply)

- I am currently on the waiting list for TKR surgery
- I have previously undergone TKR surgery

*Panellists only saw questions 10h to 10l if they selected ‘Professional’ for question 10*

10h. What region of the UK do you currently work in?

- Scotland
- Northern Ireland
- Wales
- North East
- North West
- Yorkshire and the Humber
- East Midlands
- West Midlands
- South West
- South East
- East of England
- London

10i What is your current professional role(s)? (Please select all options that apply)

- Orthopaedic surgeon
- Orthopaedic registrar
- Advanced arthroplasty practitioner
- Nurse
- Physiotherapist
- Occupational therapist
- Rehabilitation assistant
- Occupational therapy technician
- Clinical commissioner
- Manager
- Researcher
- Other (if selected, were asked to specify)

10j. How long have you worked as a health professional in years?

10k. What setting(s) do you currently work in? (Please select all options that apply)

- NHS Teaching Hospital
- NHS District / General Hospital
- NHS Community Hospital or other community location(s)
- Private Hospital or other private location(s)
- Commissioning Organisation
- University
- Other (if selected, were asked to specify)

10l. Do you currently provide clinical care to patients who are listed for or have undergone TKR surgery?

- Yes
- No

*Panellists only saw questions 10li to 10lii if they selected ‘Yes’ for question 10l*

10li. What phases of the TKR care pathway do you currently work in? (Please select all options that apply) Select ‘More info’ if you would like to see further information about these care phases *More info*

- Pre-operative phase
- Acute phase
- Post-acute phase

10lii. How many patients who are listed for TKR surgery or who have previously undergone TKR surgery did you see last week?

- < 1
- 1 – 2
- 3 – 5
- 6 – 10
- > 10

# Page 3: Pre-operative total knee replacement (TKR) education topics

This section includes an initial recommendation on topics that could be included in pre-operative education for patients waiting for total knee replacement (TKR) surgery. Please rate how important you think each individual topic is on the scale provided.

Please only rate a topic as ‘Important’ or ‘Very important’ if you think it should be included in the final list of recommendations.

It does not matter if you do not have experience of a particular topic. We would still like you to rate how important you think it is.

To see further information about each topic, select ‘More info’. If you find it difficult to see the scale on your mobile phone, select ‘view as separate questions instead’.

Recommendation 1

At a minimum, pre-operative TKR education should include the following topics:

*Background information*

- 1. Anatomy of the knee joint *More info*
  2. Health conditions that may contribute to needing TKR surgery *More info*
  3. Alternative treatment options to TKR surgery *More info*

*Preparing for TKR surgery*

- 1. Purpose of pre-operative rehabilitation *More info*
  2. Patient involvement in their own management *More info*
  3. Goal setting *More info*
  4. Using heat and cold *More info*
  5. Obtaining and using walking aids and other equipment *More info*
  6. Making home preparations *More info*
  7. Arranging social support *More info*
  8. Arranging transport to and from the hospital *More info*

*Understanding what to expect*

- 1. What to expect during the hospital stay *More info*
  2. What a TKR surgical procedure involves *More info*
  3. Risks of TKR surgery and how to minimise them *More info*
  4. Common issues that may occur following TKR surgery which do not need to cause alarm *More info*
  5. Pain expectations *More info*
  6. What to expect following discharge *More info*
  7. Recovery expectations *More info*

*Recovering from TKR surgery*

- 1. Pain management *More info*
  2. Rehabilitation following TKR surgery *More info*
  3. Complementary and alternative therapies *More info*
  4. Returning to daily activities *More info*
  5. Returning to driving and other types of travel *More info*
  6. Returning to sports and leisure activities *More info*
  7. Returning to work *More info*

*Healthy lifestyle guidance*

- 1. Physical activity *More info*
  2. Weight management *More info*
  3. Stopping smoking *More info*
  4. Avoiding alcohol misuse *More info*

If there are any additional topics you think are important to include in pre-operative TKR education, please enter them in the box below (optional)

# Page 4: Pre-operative total knee replacement (TKR) education delivery

This section includes an initial list of recommendations on the delivery of pre-operative education for patients waiting for total knee replacement (TKR) surgery. Please rate how important you think each recommendation is on the scale provided. Some recommendations include more than one option. For these recommendations, please rate how important you think each individual option is.

Please only rate a delivery approach as ‘Important’ or ‘Very important’ if you think it should be included in the final list of recommendations.

It does not matter if you do not have experience of a particular delivery approach. We would still like you to rate how important you think it is.

To see further information about certain delivery approaches, select ‘More info’. If you find it difficult to see the scale on your mobile phone, select ‘view as separate questions instead’.

Recommendation 2.1

Pre-operative TKR education should be informed by a multi-disciplinary team rather than members of a single profession *More info*

Recommendation 2.2

At a minimum, pre-operative TKR education should be informed by members of the following care teams:

- - 1. Orthopaedic surgery team *More info*
    2. Nursing team *More info*
    3. Physiotherapy team *More info*
    4. Occupational therapy team *More info*
    5. Social work team *More info*

Recommendation 2.3

Pre-operative TKR education should be informed by patients who have previously had TKR surgery

Recommendation 2.4

At least some pre-operative TKR education should be delivered by providing examples of other patients’ experiences of TKR surgery *More info*

Recommendation 2.5

At a minimum, pre-operative TKR education should be delivered using the following formats:

- - 1. Face-to-face group sessions
    2. Face-to-face individual sessions
    3. Booklet or other written format
    4. Video or DVD
    5. Website or other electronic format
    6. Telephone
    7. PowerPoint presentation *More info*

Recommendation 2.6

Pre-operative TKR education should be delivered using a combination of more than one format *More info*

Recommendation 2.7

Pre-operative TKR education should be delivered through a combination of providing the patient with information and giving them an opportunity to actively take part in tasks *More info*

Recommendation 2.8

Pre-operative TKR education should provide an opportunity for the patient’s questions to be addressed

Recommendation 2.9

Pre-operative TKR education should provide an opportunity for a family member or friend of the patient to be involved *More info*

Recommendation 2.10

Pre-operative TKR education should be tailored according to each patient’s individual needs *More info*

Recommendation 2.11

Pre-operative TKR education should be tailored according to whether the patient is having their right or their left knee replaced *More info*

Recommendation 2.12 *More info*

Patients waiting for TKR surgery should receive pre-operative education separately from patients waiting for other types of surgery, such as total hip replacement surgery *More info*

If there are any additional aspects of pre-operative TKR education delivery you think are important, please enter them in the box below (optional)

# Page 5: Pre-operative total knee replacement (TKR) exercise types

This section includes an initial recommendation on types of exercise that could be included in a pre-operative exercise programme for patients waiting for total knee replacement (TKR) surgery. Please rate how important you think each individual type of exercise is on the scale provided.

Please only rate an exercise type as ‘Important’ or ‘Very important’ if you think it should be included in the final list of recommendations.

It does not matter if you do not have direct experience of a particular type of exercise. We would still like you to rate how important you think it is.

To see further information about each type of exercise, select ‘More info’. If you find it difficult to see the scale on your mobile phone, select ‘view as separate questions instead’.

Recommendation 3

At a minimum, a pre-operative TKR exercise programme should include the following types of exercise:

*Strengthening exercises*

- 1. Leg strengthening exercises *More info*
  2. Arm strengthening exercises *More info*

*Flexibility exercises*

- 1. Leg flexibility exercises *More info*
  2. Arm flexibility exercises *More info*
  3. Torso flexibility exercises *More info*

*Functional exercises*

- 1. Balance exercises *More info*
  2. Functional movement exercises *More info*
  3. Functional technique exercises *More info*

*Other types of exercise*

- 1. Warm-up exercises *More info*
  2. Cool-down exercises *More info*
  3. Cardiovascular exercises *More info*
  4. Core control exercises *More info*
  5. Walking practice with walking aids *More info*
  6. Training on steps *More info*

If there are any additional types of exercise you think are important to include in a pre-operative TKR exercise programme, please enter them in the box below (optional)

# Page 6: Pre-operative total knee replacement (TKR) exercise programme delivery

This section includes an initial list of recommendations on the delivery of a pre-operative exercise programme for patients waiting for total knee replacement (TKR) surgery. Please rate how important you think each recommendation is on the scale provided. Some recommendations include more than one option. For these recommendations, please rate how important you think each individual option is.

Please only rate a delivery approach as ‘Important’ or ‘Very important’ if you think it should be included in the final list of recommendations.

It does not matter if you do not have experience of a particular delivery approach. We would still like you to rate how important you think it is.

To see further information about certain delivery approaches, select ‘More info’. If you find it difficult to see the scale on your mobile phone, select ‘View as separate questions instead’.

Recommendation 4.1

At a minimum, a pre-operative TKR exercise programme should be delivered using the following formats:

- - 1. Individual instruction session *More info*
    2. Supervised exercise sessions *More info*
    3. Unsupervised exercise sessions *More info*
    4. Telephone-delivered exercise sessions *More info*
    5. Booklet or other written format *More info*

Recommendation 4.2

A pre-operative TKR exercise programme should be delivered using a combination of more than one format *More info*

Recommendation 4.3

At a minimum, at least some pre-operative TKR exercise sessions should take place in the following locations:

4.3.1 The patient’s own home

4.3.2 A clinical setting, such as a hospital or GP practice

4.3.3 A community setting, such as a sports centre

Recommendation 4.4

At a minimum, a pre-operative TKR exercise programme should include exercises which are:

4.4.1 High intensity *More info*

4.4.2 Low to moderate intensity *More info*

Recommendation 4.5

A pre-operative TKR exercise programme should be tailored according to the patient’s ability *More info*

Recommendation 4.6

A pre-operative TKR exercise programme should be progressive *More info*

Recommendation 4.7

Each session in a pre-operative TKR exercise programme should last a minimum of fifteen minutes

Recommendation 4.8

A pre-operative TKR exercise programme should involve a minimum of two exercise sessions per week

Recommendation 4.9

A pre-operative TKR exercise programme should ideally be performed for a minimum of six weeks

If there are any additional aspects of pre-operative TKR exercise programme delivery you think are important, please enter them in the box below (optional)

# Page 7: Other pre-operative total knee replacement (TKR) treatments

This section includes an initial list of recommendations on other treatments that could be provided to patients waiting for total knee replacement (TKR) surgery. Please rate how important you think each recommendation is on the scale provided.

Please only rate a treatment as ‘Important’ or ‘Very important’ if you think it should be included in the final list of recommendations.

It does not matter if you do not have experience of a particular treatment. We would still like you to rate how important you think it is.

To see further information about each treatment, select ‘More info’. If you find it difficult to see the scale on your mobile phone, select ‘view as separate questions instead’.

Recommendation 5.1

Patients waiting for TKR surgery who have a BMI of 27 kg/m² or over should be referred to a weight management programme *More info*

Recommendation 5.2

Patients waiting for TKR surgery who have been formally diagnosed anxiety or depression should be offered cognitive behavioural therapy (CBT)-based therapy *More info*

Recommendation 5.3

Patients waiting for TKR surgery should be offered motivational interviewing *More info*

Recommendation 5.4

Patients waiting for TKR surgery should be offered Neuromuscular Electrical Stimulation (NMES) *More info*

Recommendation 5.5

Patients waiting for TKR surgery should be offered electroacupuncture *More info*

If there are any additional treatments you think are important to include in pre-operative TKR care, please enter them in the box below (optional)

# End of questionnaire

Thank you very much again for completing this questionnaire.

We will email you the second questionnaire towards the end of January.
