# Supplementary material for: Consensus on pre-operative total knee replacement education and prehabilitation recommendations: a UK-based modified Delphi study
Source: BMC Musculoskelet Disord. 2021 Apr 14;22:352. doi: 10.1186/s12891-021-04160-5 (PMC8044503; doi:10.1186/s12891-021-04160-5)
Supplement: Supplementary file 3 — Additional file 3: Formative categorisation matrix. Formative categorisation matrix developed from the Round 1 survey (Supplementary Table 2). [file 12891_2021_4160_MOESM3_ESM.docx]

**Consensus on pre-operative total knee replacement education and prehabilitation recommendations:**

**A UK-based modified Delphi study**

**Additional File 3: Formative categorisation matrix**

**Supplementary Table 2: Formative categorisation matrix**

| **Main category** | **Definition** | **Coding rules** | **Anchor sample** | **Potential sub-categories** |
| --- | --- | --- | --- | --- |
| 1. Pre-operative TKR education topics | Subjects which could be included in pre-operative education for patients listed for TKR surgery | Any aspects related to a specific subject that could be included in pre-operative education for patients listed for TKR surgery, excluding how the subject is delivered. Aspects related to pre-operative education in general, rather than a specific subject, are not included in this category. | *Expected length of stay at hospital – Important.* | Recommendations 1.1 – 1.29 |
| 2. Pre-operative TKR education delivery | How pre-operative education could be provided to patients listed for TKR surgery | Any aspects related to how pre-operative education could be provided to patients listed for TKR surgery, excluding the subject of the education. | *…having reading material and/or a website to refer to would be invaluable.* | Recommendations 2.1 – 2.12 |
| 3. Pre-operative TKR exercise types | Types of planned, structured physical activity which could be undertaken by patients listed for TKR surgery with the aim of increasing or maintaining their physical fitness | Any aspects related to a type of planned, structured physical activity which could be undertaken by patients listed for TKR surgery with the aim of increasing or maintaining their physical fitness, excluding how the exercise is delivered. Aspects related to pre-operative exercise in general, rather than a specific type of exercise, are not included in this category. | *Functional exercises best* | Recommendations 3.1 – 3.14 |
| 4. Pre-operative TKR exercise programme delivery | How an exercise programme could be provided to patients listed for TKR surgery | Any aspects related to how an exercise programme could be provided to patients listed for TKR surgery, excluding the type of exercise. | *I would not have been able to get time off work to attend exercise classes beforehand …* | Recommendations 4.1 – 4.9 |
| 5. Other pre-operative TKR treatments | Any interventions, other than education and exercise, which could be provided to patients listed for TKR surgery | Any aspects related to interventions, other than education and exercise, which could be provided to patients listed for TKR surgery, including how the intervention is delivered. Aspects related to pre-operative care in general, rather than a specific intervention, are not included in this category. | *Offered cognitive behavioural therapy (CBT)-based therapy – In ‘’ideal world’’ I actually think YES – but again we need evidence for this – area for research for sure.* | Recommendations 5.1 – 5.5 |

*TKR* total knee replacement

The formative categorisation matrix was developed based on the Round 1 survey.

The anchor samples were selected from panellists’ Round 1 free-text responses.
