# Supplementary material for: Consensus on pre-operative total knee replacement education and prehabilitation recommendations: a UK-based modified Delphi study
Source: BMC Musculoskelet Disord. 2021 Apr 14;22:352. doi: 10.1186/s12891-021-04160-5 (PMC8044503; doi:10.1186/s12891-021-04160-5)
Supplement: Supplementary file 4 — Additional file 4: Recruitment flow charts. Patient recruitment flow chart (Supplementary Fig. 1) and professional recruitment flow chart (Supplementary Fig. 2). [file 12891_2021_4160_MOESM4_ESM.docx]

**Consensus on pre-operative total knee replacement education and prehabilitation recommendations: A UK-based modified Delphi study**

**Additional File 4: Recruitment flow charts**


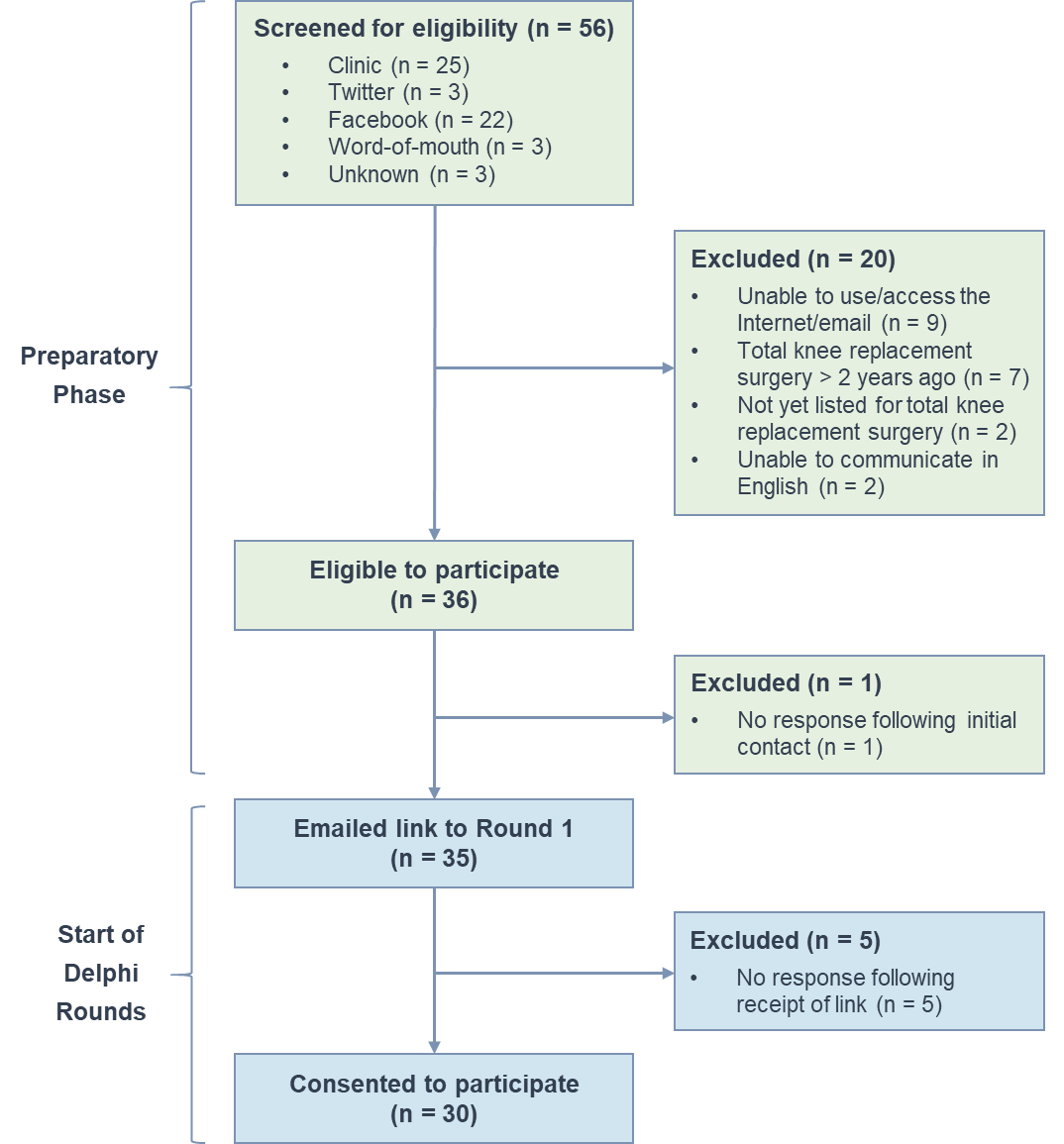


**Supplementary Figure 1: Patient recruitment flow chart**

**
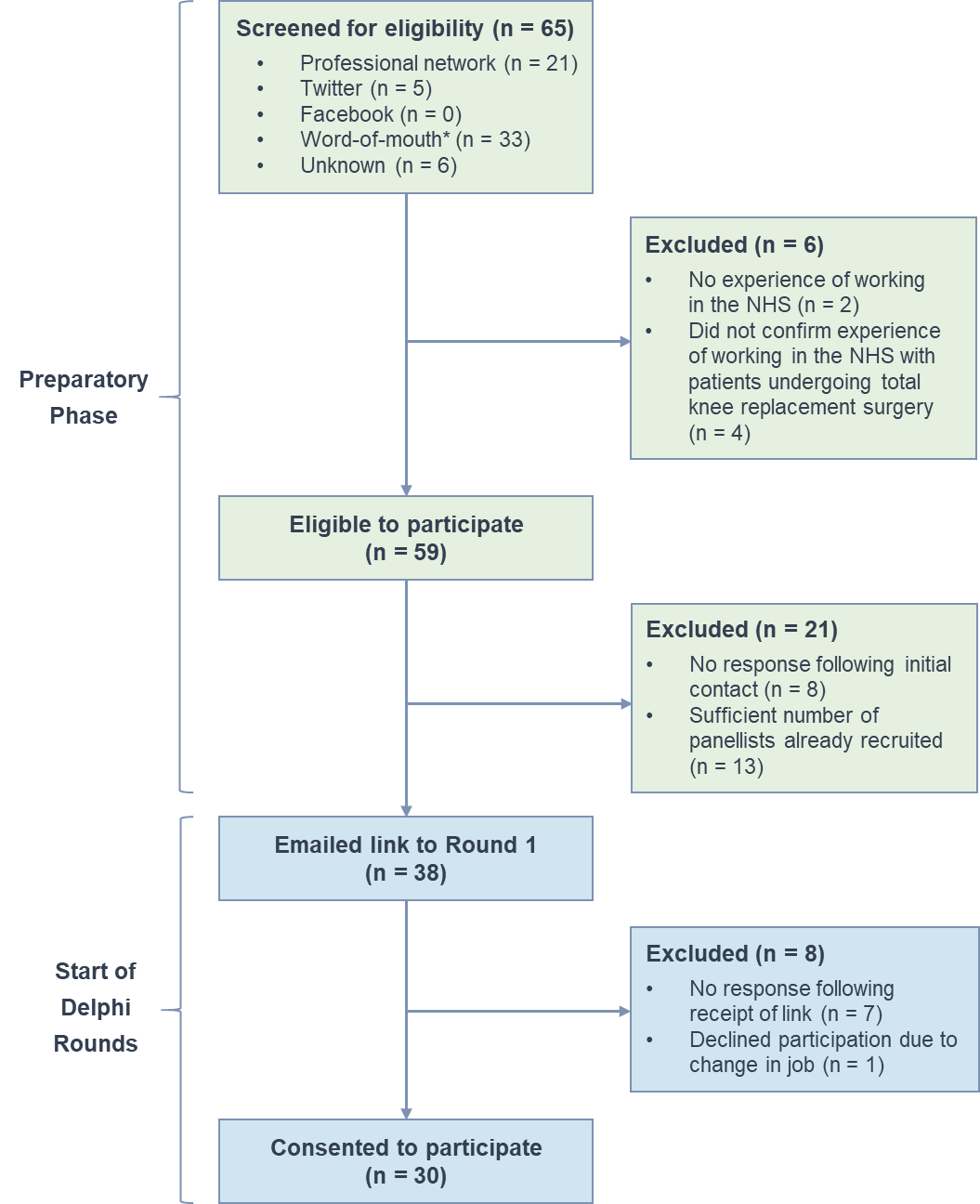
**

**Supplementary Figure 2: Professional recruitment flow chart**

*Includes professionals recruited through encouraging professionals to share the study details with other professionals
