# Supplementary material for: Consensus on pre-operative total knee replacement education and prehabilitation recommendations: a UK-based modified Delphi study
Source: BMC Musculoskelet Disord. 2021 Apr 14;22:352. doi: 10.1186/s12891-021-04160-5 (PMC8044503; doi:10.1186/s12891-021-04160-5)
Supplement: Supplementary file 5 — Additional file 5: Final categorisation matrix. Final categorisation matrix developed from the content analysis of the Round 1 free-text responses (Supplementary Table 3). [file 12891_2021_4160_MOESM5_ESM.docx]

**Consensus on pre-operative total knee replacement education and prehabilitation recommendations:**

**A UK-based modified Delphi study**

**Additional File 5: Final categorisation matrix**

**Supplementary Table 3: Final categorisation matrix**

| **Main category 1: Pre-operative TKR education topics** | | | | | |
| --- | --- | --- | --- | --- | --- |
| **Generic category** | **MU** | **Sub-category^a^** | **MU** | **Codes** | **MU** |
| Preparing for TKR surgery | 20 | 1.4 Purpose of pre-operative rehabilitation | 5 | Benefits of pre-operative exercise^b^ | 3 |
|  |  |  |  | Exercise principles | 2 |
|  |  | 1.5 Patient involvement in their own management | 2 | Importance of active participation | 2 |
|  |  | 1.8 Obtaining and using walking aids and other equipment | 1 | Walking stick | 1 |
|  |  | 1.9 Making home preparations | 3 | Need for home preparations related to other health issues | 2 |
|  |  |  |  | Importance of home preparations | 1 |
|  |  | 1.10 Arranging social support | 4 | Importance of social support | 3 |
|  |  |  |  | Need for home care related to other health issues | 1 |
|  |  | 1.11 Arranging transport to and from the hospital | 1 | Importance of planning transport | 1 |
|  |  | **1.30 Optimising management of diabetes**  **(3.3% of panellists)** | 2 | Optimising management of diabetes | 2 |
|  |  | **1.31 Education for other people, such as carers**  **(3.3% of panellists)** | 2 | Managing the expectations of others | 1 |
|  |  |  |  | Information for carers | 1 |
| Understanding what to expect | 39 | 1.12 What to expect during the hospital stay | 4 | Rehabilitation discharge criteria | 1 |
|  |  |  |  | Length of stay | 3 |
|  |  | 1.13 What a TKR surgical procedure involves | 6 | Knee prosthesis | 1 |
|  |  |  |  | Mako robot | 1 |
|  |  |  |  | Patients may not want to know about the surgical procedure | 2 |
|  |  |  |  | Anaesthetic options | 2 |
|  |  | 1.14 Risks of TKR surgery and how to minimise them | 4 | Risk of infection | 1 |
|  |  |  |  | Risk of ongoing pain | 2 |
|  |  |  |  | Risk of further interventions | 1 |
|  |  | 1.16 Pain expectations | 5 | Pain severity | 3 |
|  |  |  |  | Professionals’ pain expectations | 1 |
|  |  |  |  | Impact of pain on mobility | 1 |
|  |  | 1.17 What to expect following discharge | 2 | More information on after-care needed | 1 |
|  |  |  |  | Post-operative medication | 1 |
|  |  | 1.18 Recovery expectations | 18 | Recovery of knee function | 3 |
|  |  |  |  | Recovery of mobility | 1 |
|  |  |  |  | Differences compared to total hip replacement recovery | 2 |
|  |  |  |  | Recovery is ‘hard work’ | 1 |
|  |  |  |  | Importance of emphasising ‘everyone recovers differently’ | 2 |
|  |  |  |  | Uncertainties about recovery | 1 |
|  |  |  |  | Recovery timescales | 8 |
| Recovering from TKR surgery | 27 | 1.19 Pain management | 8 | Importance of pain medication education | 5 |
|  |  |  |  | Importance of good pain management | 3 |
|  |  | 1.20 Rehabilitation following TKR surgery | 6 | Importance of post-operative rehabilitation | 4 |
|  |  |  |  | Inadequacies of current rehabilitation | 1 |
|  |  |  |  | Rehabilitation timescales | 1 |
|  |  | 1.21 Complementary and alternative therapies | 1 | Complementary and alternative therapies uncertainties | 1 |
|  |  | 1.22 Returning to daily activities | 1 | Returning to kneeling may not be possible | 1 |
|  |  | 1.24 Returning to sports and leisure activities | 2 | Returning to high impact activities may not be possible | 1 |
|  |  |  |  | Returning to using a swimming pool | 1 |
|  |  | 1.25 Returning to work | 1 | Managing the expectations of colleagues | 1 |
|  |  | **Wound care**  **(1.7% of panellists)** | 1 | Wound care | 1 |
|  |  | **1.32 Swelling**  **(5% of panellists)** | 3 | Managing swelling | 2 |
|  |  |  |  | Swelling expectations | 1 |
|  |  | **1.33 Organising help if complications occur**  **(3.3% of panellists)** | 2 | Organising help if complications occur | 2 |
|  |  | **1.34 Gait education**  **(3.3% of panellists)** | 2 | Gait education | 2 |
| Healthy lifestyle guidance | 13 | 1.26 Physical activity | 1 | Physical activity guidance | 1 |
|  |  | 1.27 Weight management | 2 | Importance of weight management advice | 2 |
|  |  | 1.28 Stopping smoking | 2 | Smoking cessation | 2 |
|  |  | 1.29 Avoiding alcohol misuse | 1 | Avoiding alcohol | 1 |
|  |  | **1.35 Emotional well-being**  **(6.7% of panellists)** | 4 | Post-op mental impact | 2 |
|  |  |  |  | Aim of pre-operative education | 1 |
|  |  |  |  | Role of coping strategies | 1 |
|  |  | **Activity pacing**  **(1.7% of panellists)** | 2 | Activity pacing | 2 |
|  |  | **Diet and sunlight for optimising bone health**  **(1.7% of panellists)** | 1 | Diet and sunshine for optimising bone health | 1 |
| **Main category 2: Pre-operative TKR education delivery** | | | | | |
| **Generic category** | **MU** | **Sub-category^a^** | **MU** | **Codes** | **MU** |
| Roles of professional groups in pre-operative education | 12 | 2.1 Informed by a multidisciplinary team | 2 | Value of multidisciplinary input | 1 |
|  |  |  |  | Delivery by any specialist appropriate | 1 |
|  |  | 2.2.1 Informed by the orthopaedic surgery team | 1 | Respect for surgeons | 1 |
|  |  | 2.2.2 Informed by the nursing team | 3 | Nurse involvement appropriate | 3 |
|  |  | 2.2.3 Informed by the physiotherapy team | 2 | ‘Physio’ involvement useful | 2 |
|  |  | 2.2.4 Informed by the occupational therapy team | 2 | OT involvement useful | 1 |
|  |  |  |  | Surgeons may exclude OT due to discharge delay concerns | 1 |
|  |  | 2.2.5 Informed by the social work team | 1 | Social work availability issues | 1 |
|  |  | **Informed by the arthroplasty care practitioner team**  **(1.7% of panellists)** | 1 | Arthroplasty care practitioner involvement appropriate | 1 |
| Roles of previous patients in pre-operative education | 5 | 2.3 Informed by patients who have previously had TKR surgery | 1 | Potential value of involving previous patients | 1 |
|  |  | 2.4 Provide examples of other patients’ experiences of TKR surgery | 4 | Value of patients stories | 3 |
|  |  |  |  | Importance of positive and negative stories | 1 |
| Delivery formats | 24 | 2.5.1 Delivered using face-to-face group sessions | 8 | Issues with group sessions | 4 |
|  |  |  |  | Value of group sessions | 2 |
|  |  |  |  | Social support during group sessions | 2 |
|  |  | 2.5.3 Delivered using a booklet or other written format | 4 | Value of written format | 2 |
|  |  |  |  | Written format not optimal | 1 |
|  |  |  |  | Linking of written information to consent process | 1 |
|  |  | 2.5.4 Delivered using a video or DVD | 3 | Value of patient story videos | 2 |
|  |  |  |  | Patients may not want to see videos of TKR procedure | 1 |
|  |  | 2.5.5 Delivered using a website or other electronic format | 5 | Current issues with Internet access | 2 |
|  |  |  |  | Value of websites | 2 |
|  |  |  |  | Interactivity of websites | 1 |
|  |  | 2.6 Delivered using a combination of more than one format | 2 | Combining DVD/paper/electronic formats not necessary | 1 |
|  |  |  |  | Value of combining delivery formats | 1 |
|  |  | 2.7 Delivered through a combination of information provision and an opportunity to actively take part in tasks | 2 | Practicing tasks | 1 |
|  |  |  |  | Value of linking a quiz to the consent process | 1 |
| Practical arrangements | 6 | 2.9 Provide an opportunity for a family member/friend to be involved | 1 | Impact of family/friend involvement | 1 |
|  |  | **2.13 Timing of delivery**  **(6.7% of panellists)** | 5 | Importance of considering timing | 3 |
|  |  |  |  | Optimal timing | 2 |
| Setting | 3 | **2.14 Delivered in a hospital setting**  **(3.3% of panellists)** | 2 | Education delivery on ward | 2 |
|  |  | **Delivered in a community setting**  **(1.7% of panellists)** | 1 | Education delivery in leisure centres | 1 |
| Tailoring versus standardisation | 16 | 2.10 Tailored to each individual’s needs | 11 | Considerations for tailoring | 7 |
|  |  |  |  | Importance of tailoring according to patients’ needs | 3 |
|  |  |  |  | Inadequacy of current tailoring | 1 |
|  |  | 2.11 Tailored to the right or left knee | 1 | Tailoring to right or left knee ‘not at all important’ | 1 |
|  |  | 2.12 Received separately from patients waiting for other types of surgery | 2 | Some degree of tailoring to TKR important | 2 |
|  |  | **2.15 Standardisation of pre-operative education**  **(3.3% of panellists)** | 2 | Standardisation of pre-operative education | 2 |
| **Main category 3: Pre-operative TKR exercise types** | | | | | |
| **Generic category** | **MU** | **Sub-category^a^** | **MU** | **Codes** | **MU** |
| Strengthening exercises | 3 | 3.1 Leg strengthening exercises | 3 | Leg strengthening exercise examples | 1 |
|  |  |  |  | Importance of strengthening | 2 |
| Flexibility exercises | 2 | 3.3 Leg flexibility exercises | 2 | Leg flexibility exercise examples | 2 |
| Functional exercises | 6 | 3.7 Functional movement exercises | 4 | Transfer training | 2 |
|  |  |  |  | Importance of functional exercises | 2 |
|  |  | 3.6 Balance exercises | 2 | Balance exercise examples | 1 |
|  |  |  |  | Types of balance | 1 |
| Other types of exercise | 9 | 3.13 Walking practice with walking aids | 1 | Walking aid training ‘probably wasted’ pre-operatively | 1 |
|  |  | 3.14 Training on steps | 1 | Training on steps ‘probably wasted’ pre-operatively | 1 |
|  |  | **3.15 Practicing post-operative exercises**  **(3.3% of panellists)** | 2 | Practicing post-operative exercises | 2 |
|  |  | **3.16 Water-based exercises**  **(3.3% of panellists)** | 2 | Water-based exercises | 2 |
|  |  | **Open chain exercises**  **(1.7% of panellists)** | 1 | Open chain exercises | 1 |
|  |  | **3.17 Closed chain exercises**  **(3.3% of panellists)** | 2 | Closed chain exercises | 2 |
| **Main category 4: Pre-operative TKR exercise programme delivery** | | | | | |
| **Generic category** | **MU** | **Sub-category^a^** | **MU** | **Codes** | **MU** |
| Delivery format | 7 | 4.1.1 Delivered using an individual instruction session | 1 | 1:1 sessions necessary for some people | 1 |
|  |  | 4.1.2 Delivered using supervised exercise sessions | 4 | Issues with attending exercise classes | 1 |
|  |  |  |  | Value of exercise classes | 3 |
|  |  | 4.1.4 Delivered using telephone-delivered sessions | 1 | Telephone delivered sessions about ‘ticking a box’ | 1 |
|  |  | 4.2 Delivered using a combination of more than one format | 1 | Multiple formats optimal | 1 |
| Setting | 6 | 4.3.1 Take place in the patient’s own home | 4 | Value of home setting | 3 |
|  |  |  |  | Issues with home setting | 1 |
|  |  | 4.3.3 Take place in a community setting | 2 | Issues with community settings | 1 |
|  |  |  |  | Exercises for the gym | 1 |
| Tailoring | 13 | 4.5 Tailored to the patient’s ability | 3 | Importance of tailoring according to ability | 3 |
|  |  | **4.10 Tailored to each patient’s needs**  **(11.7% of panellists)** | 10 | Importance of tailoring in general | 4 |
|  |  |  |  | Considerations for tailoring | 6 |
| Exercise intensity | 4 | 4.4.1 Include high intensity exercises | 2 | Concerns about specifying intensity | 1 |
|  |  |  |  | Importance of specifying intensity | 1 |
|  |  | 4.6 Be progressive | 1 | Progress ‘as able’ | 1 |
|  |  | **Exercising both legs**  **(1.7% of panellists)** | 1 | Exercising both legs | 1 |
| Practical arrangements | 14 | 4.7 Each session should last a minimum of 15 minutes | 2 | Concerns about specifying about time | 2 |
|  |  | 4.9 Ideally be performed for a minimum of six weeks | 4 | Optimal timing | 3 |
|  |  |  |  | Limitations on exercise timing | 1 |
|  |  | **Delivered by a trained professional**  **(1.7% of panellists)** | 1 | Importance of trained professionals delivering exercises | 1 |
|  |  | **Involve family member assistance**  **(1.7% of panellists)** | 1 | Family member assistance | 1 |
|  |  | **4.11 Provide an opportunity for peer support**  **(3.3% of panellists)** | 2 | Online discussion group | 1 |
|  |  |  |  | Peer support at group sessions | 1 |
|  |  | **4.12 Include goal setting**  **(3.3% of panellists)** | 3 | Providing clinical goals | 1 |
|  |  |  |  | Setting commitment goals | 2 |
|  |  | **Availability of a comprehensive programme limited to motivated patients**  **(1.7% panellists)** | 1 | Availability of a comprehensive programme limited to motivated patients | 1 |
| **Main category 5: Other pre-operative TKR treatments** | | | | | |
| **Generic category** | **MU** | **Sub-category^a^** | **MU** | **Codes** | **MU** |
| Standard treatments | 7 | 5.1 Weight management programme (patients who have a BMI of 27 kg/m² or over) | 4 | Importance of referral being patient’s choice | 1 |
|  |  |  |  | BMI threshold for referral to a weight management programme | 2 |
|  |  |  |  | Importance of weight management support | 1 |
|  |  | 5.2 CBT-based therapy (patients who have been formally diagnosed anxiety or depression) | 1 | CBT-based therapy ideal but evidence inadequate | 1 |
|  |  | **Orthotics**  **(1.7% panellists)** | 1 | Orthotics | 1 |
|  |  | **Physiotherapy**  **(1.7% of panellists)** | 1 | Physiotherapy | 1 |
| Complementary and alternative therapies | 2 | 5.4 Neuromuscular electrical stimulation | 1 | Neuromuscular electrical stimulation uncertainties | 1 |
|  |  | 5.5 Electroacupuncture | 1 | Electroacupuncture uncertainties | 1 |
| **Main category 6: Planning and prioritising TKR care** | | | | | |
| **Generic category** | **MU** | **Sub-category** | **MU** | **Codes** | **MU** |
| Value of pre-operative interventions | 12 | Importance of appropriate pre-operative education | 8 | Importance of comprehensive pre-operative education | 3 |
|  |  |  |  | Importance of appropriate pre-operative education delivery | 1 |
|  |  |  |  | Importance of setting expectations | 4 |
|  |  | Value of pre-operative exercise | 4 | Value of pre-operative exercise | 4 |
| Concerns about pre-operative interventions | 6 | Issues with pre-operative exercise | 4 | Issues with pre-operative exercise | 4 |
|  |  | Insufficient pre-operative exercise provision | 1 | Insufficient pre-operative exercise provision | 1 |
|  |  | Non-medical interventions/coping strategies may not be needed pre-operatively | 1 | Non-medical interventions/coping strategies may not be needed pre-operatively | 1 |
| Considerations for the TKR care pathway | 9 | Importance of flexibility in the TKR care pathway | 2 | Importance of tailoring according to work commitments | 1 |
|  |  |  |  | Opportunity for patients not needing TKR to be ‘filtered out’ | 1 |
|  |  | Considerations for decision-making phase | 7 | Indication for TKR | 3 |
|  |  |  |  | Importance of decision to undergo TKR | 1 |
|  |  |  |  | Importance of conservative treatments prior to decision to undergo TKR | 3 |

*CBT* Cognitive behavioural therapy

*MU* Number of meaning units labelled with a specific code or fitting within a specific sub-category/generic category. Each meaning unit is an extract from the Round 1 free-text responses.

*OT* Occupational Therapist

*TKR* Total knee replacement

^a^ Sub-categories in bold were inductively generated and relate to a pre-operative total knee replacement intervention component or delivery approach. The percentage of panellists whose free-text responses contributed to the development of these sub-categories is provided in brackets.

^b^ Only free-text responses in the education topics section of the survey were coded as ‘Benefits of pre-operative exercise’ in the ‘Pre-operative TKR education topics’ main category. All other comments relating to the value of pre-operative exercise were coded as ‘Value of pre-operative exercise’ in the ‘Planning and prioritising TKR care’ main category.

Main categories 1-5 were pre-specified in the formative categorisation matrix (Additional File 3).

Main category 6 was inductively generated during the content analysis of panellists’ Round 1 free-text responses (Additional File 6).
