# Supplementary material for: Consensus on pre-operative total knee replacement education and prehabilitation recommendations: a UK-based modified Delphi study
Source: BMC Musculoskelet Disord. 2021 Apr 14;22:352. doi: 10.1186/s12891-021-04160-5 (PMC8044503; doi:10.1186/s12891-021-04160-5)
Supplement: Supplementary file 6 — Additional file 6: Inductively generated main category. Inductively generated main category developed during the content analysis of panellists’ Round 1 free-text responses (Supplementary Table 4). [file 12891_2021_4160_MOESM6_ESM.docx]

**Consensus on pre-operative total knee replacement education and prehabilitation recommendations:**

**A UK-based modified Delphi study**

**Additional File 6: Inductively generated main category**

**Supplementary Table 4: Inductively generated main category**

| **Main category** | **Definition** | **Coding rules** | **Anchor sample** |
| --- | --- | --- | --- |
| Planning and prioritising pre-operative TKR care | Considerations for planning and prioritising pre-operative TKR care | Any aspects related to planning and prioritising pre-operative TKR care, such as the overall value of pre-operative TKR interventions. Aspects related to a specific education topic, exercise type or intervention coded in the ‘Other pre-operative TKR treatments’ category, or to the delivery of a specific pre-operative intervention, are not included in this category. | *The activities [exercise types] mentioned are important but not at the pre op stage. You run the risk of overwhelming patients with too much information* |

*TKR* total knee replacement

The inductively generated main category was developed during the content analysis of panellists’ Round 1 free-text responses.

The anchor sample was selected from panellists’ Round 1 free-text responses.
