# Supplementary material for: Consensus on pre-operative total knee replacement education and prehabilitation recommendations: a UK-based modified Delphi study
Source: BMC Musculoskelet Disord. 2021 Apr 14;22:352. doi: 10.1186/s12891-021-04160-5 (PMC8044503; doi:10.1186/s12891-021-04160-5)
Supplement: Supplementary file 7 — Additional file 7: Round 2 new items. New items generated from panellists’ Round 1 free-text responses and included in the Round 2 survey (Supplementary Table 5). [file 12891_2021_4160_MOESM7_ESM.docx]

**Consensus on pre-operative total knee replacement education and prehabilitation recommendations: A UK-based modified Delphi study**

**Additional File 7: Round 2 new items**

**Supplementary Table 5: Round 2 new items**

| **Section** | **New items** | |
| --- | --- | --- |
| Pre-operative total knee replacement (TKR) education topics | 1.30 | Optimising management of diabetes |
|  | 1.31 | Education for other people, such as carers |
|  | 1.32 | Swelling |
|  | 1.33 | Organising help if complications occur |
|  | 1.34 | Returning to a normal walking pattern |
|  | 1.35 | Emotional well-being |
| Pre-operative total knee replacement (TKR) education delivery | 2.13 | At a minimum, at least some pre-operative TKR education should be delivered within 4 weeks of the patient’s TKR surgery |
|  | 2.14 | At a minimum, at least some pre-operative TKR education should be delivered in a hospital setting, such as a ward |
|  | 2.15 | Pre-operative TKR education should be standardised across the United Kingdom |
| Pre-operative total knee replacement (TKR) exercise types | 3.15 | Practicing post-operative exercises |
|  | 3.16 | Water-based exercises |
|  | 3.17 | Exercises in which the foot does not move |
| Pre-operative total knee replacement (TKR) exercise programme delivery | 4.10 | A pre-operative TKR exercise programme should be tailored according to each patient’s individual needs |
|  | 4.11 | A pre-operative TKR exercise programme should provide an opportunity for peer support |
|  | 4.12 | A pre-operative TKR exercise programme should include goal setting |

*TKR* total knee replacement
