# Supplementary material for: Consensus on pre-operative total knee replacement education and prehabilitation recommendations: a UK-based modified Delphi study
Source: BMC Musculoskelet Disord. 2021 Apr 14;22:352. doi: 10.1186/s12891-021-04160-5 (PMC8044503; doi:10.1186/s12891-021-04160-5)
Supplement: Supplementary file 8 — Additional file 8. Final list of recommendations. Final list of recommendations developed from the Round 3 results. [file 12891_2021_4160_MOESM8_ESM.pdf]

Pre-operative total knee replacement care recommendations

This document provides recommendations on pre-operative care for patients waiting for total knee replacement (TKR) surgery in the United Kingdom.

The recommendations were developed using an approach known as a Delphi Consensus Technique. This involved a group of patients and professionals, known as ‘panel members’, completing three online questionnaires. All the items included in this list of recommendations were rated as ‘Important’ or ‘Very important’ by at least 70% of panel members in the final questionnaire.

Further details about the study used to develop the recommendations are available at: https://doi.org/10.1186/s12891-021-04160-5. A prioritised list of recommendations are available via the same link in Additional File 10.

Authors: Anna M. Anderson, Christine Comer, Toby O. Smith, Benjamin T. Drew, Hemant Pandit, Deborah Antcliff, Anthony C. Redmond, Gretl A. McHugh

This document presents independent research supported by the National Institute for Health Research (NIHR) Leeds Biomedical Research Centre (BRC). Professor Redmond and Professor Pandit are National Institute for Health Research (NIHR) Senior Investigators. Anna Anderson, Clinical Doctoral Research Fellow, ICA-CDRF-2018-04-ST2-006, is funded by Health Education England (HEE) / National Institute for Health Research (NIHR) for this research project. The views expressed in this publication are those of the author(s) and not necessarily those of the NIHR, NHS or the UK Department of Health and Social Care.

NHS The Leeds Teaching Hospitals NHS Trust

FUNDED BY NIHR | National Institute for Health Research

UNIVERSITY OF LEEDS

Pre-operative education topics

At a minimum, pre-operative total knee replacement (TKR) education should include the following topics:

Background information

- Anatomy of the knee joint
- Health conditions that may contribute to needing TKR surgery
- Alternative treatment options to TKR surgery

Preparing for TKR surgery

- Purpose of pre-operative rehabilitation
- Patient involvement in their own management
- Goal setting
- Using heat and cold
- Obtaining and using walking aids and other equipment
- Making home preparations
- Arranging social support
- Arranging transport to and from the hospital
- Optimising management of diabetes
- Emotional well-being
- Education for other people, such as carers

Understanding what to expect

- What to expect during the hospital stay
- What a TKR surgical procedure involves
- Risks of TKR surgery and how to minimise them
- Common issues that may occur following TKR surgery which do not need to cause alarm
- Pain expectations
- Swelling
- What to expect following discharge
- Recovery expectations

Recovering from TKR surgery

- Organising help if complications occur
- Pain management
- Rehabilitation following TKR surgery
- Returning to daily activities
- Returning to a normal walking pattern
- Returning to driving and other types of travel
- Returning to sports and leisure activities
- Returning to work

Healthy lifestyle guidance

- Physical activity
- Weight management
- Stopping smoking
- Avoiding alcohol misuse

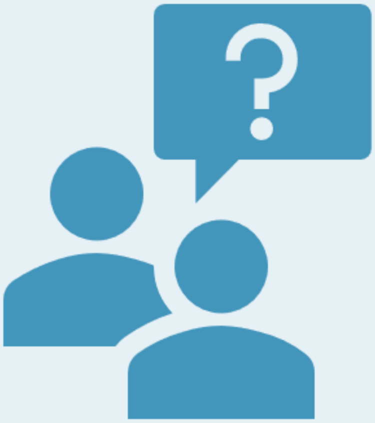

Pre-operative education delivery

Pre-operative total knee replacement (TKR) education should:

Providers

- Be informed by a multi-disciplinary team, including members of the orthopaedic surgery, nursing, physiotherapy and occupational therapy teams
- Be informed by patients who have previously had TKR surgery

Delivery mode

- Be delivered, at least partly, by providing examples of other patients' experiences of TKR surgery
- Be delivered using a combination of more than one format, including face-to-face group sessions, a booklet or other written format and a website or other electronic format
- Be delivered through a combination of providing the patient with information and giving them an opportunity to actively take part in tasks
- Be delivered to patients waiting for TKR surgery separately from education delivered to patients waiting for other types of surgery, such as total hip replacement surgery
- Provide an opportunity for the patient's questions to be addressed
- Provide an opportunity for a family member or friend of the patient to be involved

Schedule

- Be delivered, at least partly, within four weeks of the patient's TKR surgery

Tailoring

- Be tailored according to each patient's individual needs
- Be standardised across the United Kingdom

Pre-operative exercise types

At a minimum, a pre-operative TKR exercise programme should include the following types of exercise:

- Leg strengthening exercises
- Leg flexibility exercises
- Balance exercises
- Functional movement exercises
- Functional technique exercises
- Cardiovascular exercises
- Core control exercises
- Walking practice with walking aids
- Training on steps
- Practicing post-operative exercises

Pre-operative exercise delivery

A pre-operative total knee replacement (TKR) exercise programme should:

Delivery mode

- Be delivered using a combination of more than one format, including supervised exercise sessions, unsupervised exercise sessions and a booklet or other written format
- Provide an opportunity for peer support
- Include goal setting

Intensity

- Include exercises which are low to moderate intensity
- Be progressive

Schedule

- Involve exercise sessions which last a minimum of fifteen minutes each
- Involve a minimum of two exercise sessions per week
- Ideally be performed for a minimum of six weeks

Tailoring

- Be tailored according to each patient's individual needs and ability

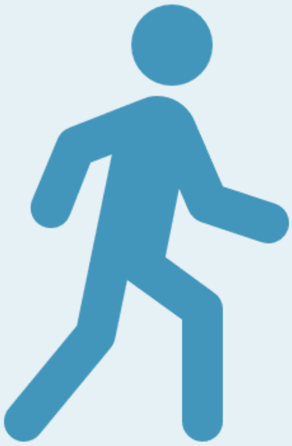

Other pre-operative treatments

Patients waiting for TKR surgery who have a body mass index (BMI) of 27 kg/m² or over should be offered referral to a weight management programme

Patients waiting for TKR surgery who have been formally diagnosed with anxiety or depression should be offered referral to cognitive behavioural therapy (CBT)-based therapy
