# Supplementary material for: Consensus on pre-operative total knee replacement education and prehabilitation recommendations: a UK-based modified Delphi study
Source: BMC Musculoskelet Disord. 2021 Apr 14;22:352. doi: 10.1186/s12891-021-04160-5 (PMC8044503; doi:10.1186/s12891-021-04160-5)
Supplement: Supplementary file 9 — Additional file 9: Detailed importance ratings results. Detailed importance rating results for Round 1 (Supplementary Tables 6–10), Round 2 (Supplementary Tables 11–15) and Round 3 (Supplementary Tables 16–20). [file 12891_2021_4160_MOESM9_ESM.docx]

**Consensus on pre-operative total knee replacement education and prehabilitation recommendations:**

**A UK-based modified Delphi study**

**Additional File 9: Detailed importance ratings results**

# Round 1

## Supplementary Table 6: Pre-operative total knee replacement education topics: Round 1 importance ratings

| **Pre-operative total knee replacement education topic item^a^** | **All panellists (n = 60)** | | | **Patient panellists (n = 30)** | | | **Professional panellists (n = 30)** | | |
| --- | --- | --- | --- | --- | --- | --- | --- | --- | --- |
|  | **% Important or Very Important rating** | **Median** | **Interquartile range** | **% Important or Very Important rating** | **Median** | **Interquartile range** | **% Important or Very Important rating** | **Median** | **Interquartile range** |
| 1.1 Anatomy of the knee joint | 77 | 4 | 0 | 70 | 4 | 1.25 | 83 | 4 | 0 |
| 1.2 Health conditions that may contribute to needing TKR surgery | 77 | 4 | 1 | 67 | 4 | 2 | 87 | 4 | 1 |
| 1.3 Alternative treatment options to TKR surgery | 82 | 4 | 1 | 77 | 4 | 0.5 | 87 | 5 | 1 |
| 1.4 Purpose of pre-operative rehabilitation | 98 | 5 | 1 | 100 | 5 | 1 | 97 | 5 | 0 |
| 1.5 Patient involvement in their own management | 98 | 5 | 0 | 97 | 5 | 1 | 100 | 5 | 0 |
| 1.6 Goal setting | 88 | 4.5 | 1 | 80 | 4 | 1 | 97 | 5 | 1 |
| 1.7 Using heat and cold | 87 | 4 | 1 | 97 | 4.5 | 1 | 77 | 4 | 0.25 |
| 1.8 Obtaining and using walking aids and other equipment | 95 | 5 | 1 | 97 | 5 | 1 | 93 | 5 | 1 |
| 1.9 Making home preparations | 98 | 5 | 1 | 97 | 4 | 1 | 100 | 5 | 1 |
| 1.10 Arranging social support | 88 | 4 | 1 | 80 | 4 | 0.25 | 97 | 5 | 1 |
| 1.11 Arranging transport to and from the hospital | 82 | 4 | 1 | 70 | 4 | 2 | 93 | 4 | 1 |
| 1.12 What to expect during the hospital stay | 98 | 5 | 0.75 | 97 | 5 | 1 | 100 | 5 | 0 |
| 1.13 What a TKR surgical procedure involves | 92 | 5 | 1 | 90 | 5 | 1 | 93 | 4 | 1 |
| 1.14 Risks of TKR surgery and how to minimise them | 97 | 5 | 1 | 97 | 5 | 1 | 97 | 5 | 1 |
| 1.15 Common issues that may occur following TKR surgery which do not need to cause alarm | 93 | 5 | 1 | 87 | 4 | 1 | 100 | 5 | 1 |
| 1.16 Pain expectations | 97 | 5 | 0 | 93 | 5 | 1 | 100 | 5 | 0 |
| 1.17 What to expect following discharge | 95 | 5 | 1 | 97 | 5 | 1 | 93 | 5 | 1 |
| 1.18 Recovery expectations | 98 | 5 | 1 | 97 | 4.5 | 1 | 100 | 5 | 0 |
| 1.19 Pain management | 100 | 5 | 0 | 100 | 5 | 1 | 100 | 5 | 0 |
| 1.20 Rehabilitation following TKR surgery | 100 | 5 | 0 | 100 | 5 | 1 | 100 | 5 | 0 |
| *1.21 Complementary and alternative therapies* | 28 | 3 | 2 | 40 | 3 | 1 | 17 | 3 | 1 |
| 1.22 Returning to daily activities | 93 | 4 | 1 | 90 | 4 | 1 | 97 | 5 | 1 |
| 1.23 Returning to driving and other types of travel | 95 | 4.5 | 1 | 93 | 4 | 1 | 97 | 5 | 1 |
| 1.24 Returning to sports and leisure activities | 90 | 4 | 1 | 87 | 4 | 0.25 | 93 | 4 | 1 |
| 1.25 Returning to work | 88 | 4 | 1 | 80 | 4 | 1 | 97 | 5 | 1 |
| 1.26 Physical activity | 95 | 5 | 1 | 90 | 4 | 1 | 100 | 5 | 1 |
| 1.27 Weight management | 90 | 4 | 1 | 83 | 4 | 1 | 97 | 5 | 1 |
| 1.28 Stopping smoking | 80 | 5 | 1 | 67 | 4 | 2 | 93 | 5 | 1 |
| 1.29 Avoiding alcohol misuse | 73 | 4 | 2 | 70 | 4 | 2 | 77 | 4 | 1.25 |

*TKR* Total knee replacement

^a^ Item in italics did not reach consensus. Consensus was defined as at least 70% of respondents rating an item as ‘Important’ or ‘Very important’.

## Supplementary Table 7: Pre-operative total knee replacement education delivery: Round 1 importance ratings

| **Pre-operative total knee replacement education delivery item^a^** | **All panellists (n = 60)** | | | **Patient panellists (n = 30)** | | | **Professional panellists (n = 30)** | | |
| --- | --- | --- | --- | --- | --- | --- | --- | --- | --- |
|  | **% Important or Very Important rating** | **Median** | **Interquartile range** | **% Important or Very Important rating** | **Median** | **Interquartile range** | **% Important or Very Important rating** | **Median** | **Interquartile range** |
| 2.1 Informed by a multidisciplinary team | 75 | 4 | 1.75 | 67 | 4 | 2 | 83 | 4 | 1 |
| 2.2.1 Informed by the orthopaedic surgery team | 82 | 4 | 1 | 87 | 4 | 1 | 77 | 4 | 1.25 |
| 2.2.2 Informed by the nursing team | 72 | 4 | 2 | 67 | 4 | 1.25 | 77 | 4 | 1.25 |
| 2.2.3 Informed by the physiotherapy team | 95 | 5 | 1 | 93 | 4 | 1 | 97 | 5 | 1 |
| 2.2.4 Informed by the occupational therapy team | 80 | 4 | 1 | 83 | 4 | 0.25 | 77 | 4 | 1.25 |
| *2.2.5 Informed by the social work team* | 42 | 3 | 1 | 47 | 3 | 1 | 37 | 3 | 1 |
| *2.3 Informed by patients who have previously had TKR surgery* | 63 | 4 | 1 | 50 | 3.5 | 1.25 | 77 | 4 | 0.5 |
| 2.4 Provide examples of other patients’ experiences of TKR surgery | 70 | 4 | 2 | 63 | 4 | 1 | 77 | 4 | 1.25 |
| 2.5.1 Delivered using face-to-face group sessions | 78 | 4 | 1 | 70 | 4 | 1 | 87 | 4.5 | 1 |
| *2.5.2 Delivered using face-to-face individual sessions* | 45 | 3 | 2 | 50 | 3.5 | 2 | 40 | 3 | 2 |
| 2.5.3 Delivered using a booklet or other written format | 88 | 4 | 1 | 87 | 4 | 1 | 90 | 4 | 1 |
| *2.5.4 Delivered using a video or DVD* | 63 | 4 | 1 | 53 | 4 | 1 | 73 | 4 | 1 |
| 2.5.5 Delivered using a website or other electronic format | 72 | 4 | 1 | 67 | 4 | 1 | 77 | 4 | 1.25 |
| *2.5.6 Delivered using telephone* | 25 | 3 | 1.75 | 17 | 3 | 1.25 | 33 | 3 | 2 |
| *2.5.7 Delivered using a PowerPoint presentation* | 32 | 3 | 2 | 27 | 3 | 2 | 37 | 3 | 1 |
| 2.6 Delivered using a combination of more than one format | 87 | 4 | 1 | 80 | 4 | 0.25 | 93 | 5 | 1 |
| 2.7 Delivered through a combination of information provision and an opportunity to actively take part in tasks | 78 | 4 | 1 | 63 | 4 | 1 | 93 | 4 | 1 |
| 2.8 Provide an opportunity for questions to be addressed | 97 | 5 | 1 | 93 | 4 | 1 | 100 | 5 | 0 |
| 2.9 Provide an opportunity for a family member/ friend to be involved | 80 | 4 | 1 | 63 | 4 | 1 | 97 | 5 | 1 |
| 2.10 Tailored to each patient’s needs | 85 | 4 | 1 | 77 | 4 | 1.25 | 93 | 4 | 1 |
| *2.11 Tailored to the right or left knee* | 13 | 2 | 2 | 23 | 3 | 1.25 | 3 | 2 | 2 |
| *2.12 Received separately from patients waiting for other types of surgery* | 67 | 4 | 2 | 70 | 4 | 1.25 | 63 | 4 | 2 |

*TKR* Total knee replacement

^a^ Items in italics did not reach consensus. Consensus was defined as at least 70% of respondents rating an item as ‘Important’ or ‘Very important’.

Additional Files 2 and 7 provide the exact wording of each item.

## Supplementary Table 8: Pre-operative total knee replacement exercise types: Round 1 importance ratings

| **Pre-operative total knee replacement exercise type item^a^** | **All panellists (n = 60)** | | | **Patient panellists (n = 30)** | | | **Professional panellists (n = 30)** | | |
| --- | --- | --- | --- | --- | --- | --- | --- | --- | --- |
|  | **% Important or Very Important rating** | **Median** | **Interquartile range** | **% Important or Very Important rating** | **Median** | **Interquartile range** | **% Important or Very Important rating** | **Median** | **Interquartile range** |
| 3.1 Leg strengthening exercises | 98 | 5 | 1 | 97 | 5 | 1 | 100 | 5 | 1 |
| *3.2 Arm strengthening exercises* | 43 | 3 | 1 | 37 | 3 | 1.25 | 50 | 3.5 | 1 |
| 3.3 Leg flexibility exercises | 83 | 4 | 1 | 87 | 4.5 | 1 | 80 | 4 | 1 |
| *3.4 Arm flexibility exercises* | 27 | 3 | 1 | 23 | 3 | 0.5 | 30 | 3 | 1.25 |
| *3.5 Torso flexibility exercises* | 43 | 3 | 1 | 43 | 3 | 1 | 43 | 3 | 1 |
| 3.6 Balance exercises | 85 | 4 | 1 | 83 | 4 | 1 | 87 | 5 | 1 |
| 3.7 Functional movement exercises | 87 | 4 | 1 | 80 | 4 | 1 | 93 | 5 | 1 |
| 3.8 Functional technique exercises | 80 | 4 | 1 | 73 | 4 | 2 | 87 | 4 | 1 |
| *3.9 Warm-up exercises* | 58 | 4 | 1 | 60 | 4 | 1 | 57 | 4 | 1 |
| *3.10 Cool-down exercises* | 48 | 3 | 1 | 53 | 4 | 1 | 43 | 3 | 1 |
| *3.11 Cardiovascular exercises* | 60 | 4 | 1 | 43 | 3 | 1 | 77 | 4 | 0.25 |
| *3.12 Core control exercises* | 60 | 4 | 1 | 53 | 4 | 1 | 67 | 4 | 1 |
| 3.13 Walking practice with walking aids | 83 | 4 | 1 | 77 | 4 | 1.25 | 90 | 5 | 1 |
| 3.14 Training on steps | 83 | 4 | 1 | 83 | 4 | 1 | 83 | 5 | 1 |

^a^ Items in italics did not reach consensus. Consensus was defined as at least 70% of respondents rating an item as ‘Important’ or ‘Very important’.

## Supplementary Table 9: Pre-operative total knee replacement exercise programme delivery: Round 1 importance ratings

| **Pre-operative total knee replacement exercise programme delivery item^a^** | **All panellists (n = 60)** | | | **Patient panellists (n = 30)** | | | **Professional panellists (n = 30)** | | |
| --- | --- | --- | --- | --- | --- | --- | --- | --- | --- |
|  | **% Important or Very Important rating** | **Median** | **Interquartile range** | **% Important or Very Important rating** | **Median** | **Interquartile range** | **% Important or Very Important rating** | **Median** | **Interquartile range** |
| *4.1.1 Delivered using an individual instruction session* | 52 | 4 | 1 | 53 | 4 | 1.25 | 50 | 3.5 | 1 |
| 4.1.2 Delivered using supervised exercise sessions | 73 | 4 | 1 | 80 | 4 | 0.25 | 67 | 4 | 1.25 |
| *4.1.3 Delivered using unsupervised exercise sessions* | 58 | 4 | 1 | 50 | 3.5 | 2 | 67 | 4 | 1 |
| *4.1.4 Delivered using telephone- delivered sessions* | 5 | 2.5 | 1 | 3 | 2 | 1 | 7 | 3 | 1 |
| 4.1.5 Delivered using a booklet or other written format | 87 | 4 | 1 | 87 | 4 | 0 | 87 | 4 | 1 |
| 4.2 Delivered using a combination of more than one format | 87 | 4 | 1 | 80 | 4 | 1 | 93 | 4 | 1 |
| *4.3.1 Take place in the patient’s own home* | 53 | 4 | 3 | 47 | 3 | 2 | 60 | 4 | 3 |
| *4.3.2 Take place in a clinical setting* | 52 | 4 | 1 | 57 | 4 | 1 | 47 | 3 | 1 |
| *4.3.3 Take place in a community setting* | 52 | 4 | 1 | 47 | 3 | 2 | 57 | 4 | 1 |
| *4.4.1 Include high intensity exercises* | 33 | 3 | 2 | 50 | 3.5 | 2 | 17 | 3 | 1 |
| 4.4.2 Include low to moderate intensity exercises | 75 | 4 | 0.75 | 70 | 4 | 1 | 80 | 4 | 0 |
| 4.5 Tailored to the patient’s ability | 93 | 4 | 1 | 87 | 4 | 1 | 100 | 5 | 1 |
| 4.6 Be progressive | 82 | 4 | 1 | 83 | 4 | 0.25 | 80 | 4 | 1 |
| *4.7 Each session should last a minimum of 15 minutes* | 63 | 4 | 2 | 70 | 4 | 2 | 57 | 4 | 2 |
| 4.8 Involve a minimum of 2 sessions per week | 78 | 4 | 1 | 73 | 4 | 2 | 83 | 4 | 1 |
| 4.9 Ideally be performed for minimum of 6 weeks | 80 | 4 | 1 | 77 | 4 | 1.25 | 83 | 4 | 1 |

^a^ Items in italics did not reach consensus. Consensus was defined as at least 70% of respondents rating an item as ‘Important’ or ‘Very important’.

Additional Files 2 and 7 provide the exact wording of each item.

## Supplementary Table 10: Other pre-operative total knee replacement treatments: Round 1 importance ratings

| **Other pre-operative total knee replacement treatment item^a^** | **All panellists (n = 60)** | | | **Patient panellists (n = 30)** | | | **Professional panellists (n = 30)** | | |
| --- | --- | --- | --- | --- | --- | --- | --- | --- | --- |
|  | **% Important or Very Important rating** | **Median** | **Interquartile range** | **% Important or Very Important rating** | **Median** | **Interquartile range** | **% Important or Very Important rating** | **Median** | **Interquartile range** |
| *5.1 Patients who have a BMI of 27 kg/m² or over should be referred to a weight management programme* | 67 | 4 | 1 | 53 | 4 | 1 | 80 | 4 | 1 |
| *5.2 Patients who have been formally diagnosed anxiety or depression should be offered CBT-based therapy* | 67 | 4 | 1 | 60 | 4 | 1 | 73 | 4 | 2 |
| *5.3 Patients should be offered motivational interviewing* | 38 | 3 | 1 | 23 | 3 | 1.25 | 53 | 4 | 1 |
| *5.4 Patients should be offered neuromuscular electrical stimulation* | 17 | 3 | 1 | 27 | 3 | 2 | 7 | 2 | 2 |
| *5.5 Patients should be offered electro-acupuncture* | 8 | 2 | 2 | 10 | 3 | 1.25 | 7 | 2 | 2 |

*BMI* Body Mass Index

*CBT* Cognitive behavioural therapy

^a^ Items in italics did not reach consensus. Consensus was defined as at least 70% of respondents rating an item as ‘Important’ or ‘Very important’.

Additional File 2 provides the exact wording of each item.

# Round 2

## Supplementary Table 11: Pre-operative total knee replacement education topics: Round 2 importance ratings

| **Pre-operative total knee replacement education topic item^a^** | **All panellists (n = 57)** | | | **Patient panellists (n = 28)** | | | **Professional panellists (n = 29)** | | |
| --- | --- | --- | --- | --- | --- | --- | --- | --- | --- |
|  | **% Important or Very Important rating** | **Median** | **Interquartile range** | **% Important or Very Important rating** | **Median** | **Interquartile range** | **% Important or Very Important rating** | **Median** | **Interquartile range** |
| 1.1 Anatomy of the knee joint | 91 | 4 | 0 | 82 | 4 | 0 | 100 | 4 | 0 |
| 1.2 Health conditions that may contribute to needing TKR surgery | 95 | 4 | 1 | 93 | 4 | 1 | 97 | 4 | 1 |
| 1.3 Alternative treatment options to TKR surgery | 91 | 5 | 1 | 93 | 4 | 1 | 90 | 5 | 1 |
| 1.4 Purpose of pre-operative rehabilitation | 98 | 5 | 0 | 96 | 5 | 0 | 100 | 5 | 0 |
| 1.5 Patient involvement in their own management | 100 | 5 | 0 | 100 | 5 | 0 | 100 | 5 | 0 |
| 1.6 Goal setting | 93 | 4 | 1 | 89 | 4 | 1 | 97 | 5 | 1 |
| 1.7 Using heat and cold | 88 | 4 | 0 | 96 | 4 | 1 | 79 | 4 | 0 |
| 1.8 Obtaining and using walking aids and other equipment | 96 | 5 | 1 | 96 | 5 | 0.75 | 97 | 5 | 1 |
| 1.9 Making home preparations | 98 | 5 | 0.5 | 96 | 5 | 1 | 100 | 5 | 0 |
| 1.10 Arranging social support | 95 | 5 | 1 | 89 | 4 | 1 | 100 | 5 | 1 |
| 1.11 Arranging transport to and from the hospital | 91 | 4 | 1 | 89 | 4.5 | 1 | 93 | 4 | 1 |
| 1.12 What to expect during the hospital stay | 100 | 5 | 0 | 100 | 5 | 0 | 100 | 5 | 0 |
| 1.13 What a TKR surgical procedure involves | 89 | 4 | 1 | 82 | 5 | 1 | 97 | 4 | 1 |
| 1.14 Risks of TKR surgery and how to minimise them | 100 | 5 | 0 | 100 | 5 | 0 | 100 | 5 | 0 |
| 1.15 Common issues that may occur following TKR surgery which do not need to cause alarm | 98 | 5 | 1 | 96 | 5 | 1 | 100 | 5 | 0 |
| 1.16 Pain expectations | 100 | 5 | 0 | 100 | 5 | 0 | 100 | 5 | 0 |
| 1.17 What to expect following discharge | 98 | 5 | 0 | 100 | 5 | 0 | 97 | 5 | 0 |
| 1.18 Recovery expectations | 98 | 5 | 0 | 96 | 5 | 0 | 100 | 5 | 0 |
| 1.19 Pain management | 100 | 5 | 0 | 100 | 5 | 0 | 100 | 5 | 0 |
| 1.20 Rehabilitation following TKR surgery | 100 | 5 | 0 | 100 | 5 | 0 | 100 | 5 | 0 |
| *1.21 Complementary and alternative therapies* | 21 | 3 | 0 | 29 | 3 | 1 | 14 | 3 | 1 |
| 1.22 Returning to daily activities | 100 | 4 | 1 | 100 | 4 | 1 | 100 | 5 | 1 |
| 1.23 Returning to driving and other types of travel | 96 | 4 | 1 | 93 | 5 | 1 | 100 | 4 | 1 |
| 1.24 Returning to sports and leisure activities | 89 | 4 | 1 | 82 | 4 | 1 | 97 | 4 | 1 |
| 1.25 Returning to work | 95 | 4 | 1 | 89 | 4 | 1 | 100 | 5 | 1 |
| 1.26 Physical activity | 98 | 5 | 1 | 100 | 4.5 | 1 | 97 | 5 | 1 |
| 1.27 Weight management | 98 | 5 | 1 | 96 | 4 | 1 | 100 | 5 | 1 |
| 1.28 Stopping smoking | 84 | 5 | 1 | 71 | 5 | 2 | 97 | 5 | 1 |
| 1.29 Avoiding alcohol misuse | 82 | 4 | 0 | 79 | 4 | 0 | 86 | 4 | 0.5 |
| 1.30 Optimising management of diabetes | 77 | 4 | 1 | 75 | 4 | 1.5 | 79 | 4 | 1 |
| 1.31 Education for other people, such as carers | 82 | 4 | 1 | 75 | 4 | 0.75 | 90 | 4 | 1 |
| 1.32 Swelling | 98 | 5 | 1 | 96 | 4 | 1 | 100 | 5 | 1 |
| 1.33 Organising help if complications occur | 100 | 5 | 1 | 100 | 4.5 | 1 | 100 | 5 | 1 |
| 1.34 Returning to a normal walking pattern | 93 | 4 | 1 | 100 | 4.5 | 1 | 86 | 4 | 1 |
| 1.35 Emotional well-being | 89 | 4 | 1 | 89 | 4 | 1 | 90 | 4 | 1 |

*TKR* Total knee replacement

^a^ Item in italics did not reach consensus. Consensus was defined as at least 70% of respondents rating an item as ‘Important’ or ‘Very important’.

## Supplementary Table 12: Pre-operative total knee replacement education delivery: Round 2 importance ratings

| **Pre-operative total knee replacement education delivery item^a^** | **All panellists (n = 57)** | | | **Patient panellists (n = 28)** | | | **Professional panellists (n = 29)** | | |
| --- | --- | --- | --- | --- | --- | --- | --- | --- | --- |
|  | **% Important or Very Important rating** | **Median** | **Interquartile range** | **% Important or Very Important rating** | **Median** | **Interquartile range** | **% Important or Very Important rating** | **Median** | **Interquartile range** |
| 2.1 Informed by a multidisciplinary team | 91 | 4 | 1 | 86 | 4 | 1 | 97 | 5 | 1 |
| 2.2.1 Informed by the orthopaedic surgery team | 93 | 4 | 1 | 89 | 4 | 1 | 97 | 5 | 1 |
| 2.2.2 Informed by the nursing team | 86 | 4 | 1 | 82 | 4 | 1 | 90 | 5 | 1 |
| 2.2.3 Informed by the physiotherapy team | 98 | 5 | 0 | 96 | 5 | 1 | 100 | 5 | 0 |
| 2.2.4 Informed by the occupational therapy team | 88 | 4 | 1 | 86 | 4 | 0 | 90 | 4 | 1 |
| *2.2.5 Informed by the social work team* | 42 | 3 | 1 | 43 | 3 | 1 | 41 | 3 | 1 |
| *2.3 Informed by patients who have previously had TKR surgery* | 67 | 4 | 1 | 61 | 4 | 1 | 72 | 4 | 1 |
| 2.4 Provide examples of other patients’ experiences of TKR surgery | 77 | 4 | 0.5 | 86 | 4 | 0 | 69 | 4 | 2 |
| 2.5.1 Delivered using face-to-face group sessions | 89 | 4 | 1 | 86 | 4 | 0 | 93 | 5 | 1 |
| *2.5.2 Delivered using face-to-face individual sessions* | 56 | 4 | 1 | 57 | 4 | 1 | 55 | 4 | 1 |
| 2.5.3 Delivered using a booklet or other written format | 98 | 4 | 1 | 100 | 4 | 1 | 97 | 5 | 1 |
| *2.5.4 Delivered using video or DVD* | 65 | 4 | 1 | 68 | 4 | 1 | 62 | 4 | 1 |
| 2.5.5 Delivered using a website or other electronic format | 74 | 4 | 1 | 71 | 4 | 1 | 76 | 4 | 0.5 |
| *2.5.6 Delivered using telephone* | 18 | 3 | 0 | 18 | 3 | 0 | 17 | 3 | 0 |
| *2.5.7 Delivered using a PowerPoint presentation* | 23 | 3 | 0 | 21 | 3 | 0 | 24 | 3 | 0.5 |
| 2.6 Delivered using a combination of more than one format | 93 | 5 | 1 | 89 | 4 | 1 | 97 | 5 | 1 |
| 2.7 Delivered through a combination of information provision and an opportunity to actively take part in tasks | 95 | 4 | 1 | 100 | 4 | 0 | 90 | 4 | 1 |
| 2.8 Provide an opportunity for questions to be addressed | 100 | 5 | 0 | 100 | 5 | 0.75 | 100 | 5 | 0 |
| 2.9 Provide an opportunity for a family member/ friend to be involved | 91 | 4 | 1 | 89 | 4 | 0 | 93 | 4 | 1 |
| 2.10 Tailored to each patient’s needs | 82 | 4 | 1 | 82 | 4 | 1 | 83 | 4 | 1 |
| *2.11 Tailored to the right or left knee* | 7 | 2 | 2 | 11 | 2.5 | 2 | 3 | 2 | 2 |
| 2.12 Received separately from patients waiting for other types of surgery | 75 | 4 | 1.5 | 82 | 4 | 1 | 69 | 4 | 2 |
| 2.13 Delivered within 4 weeks of TKR surgery | 79 | 4 | 1 | 82 | 4 | 1 | 76 | 4 | 1.5 |
| *2.14 Delivered in a hospital setting* | 33 | 3 | 2 | 32 | 3 | 2 | 34 | 3 | 1.5 |
| 2.15 Standardised across the UK | 72 | 4 | 1 | 75 | 4 | 1.5 | 69 | 4 | 1 |

*TKR* Total knee replacement

^a^ Items in italics did not reach consensus. Consensus was defined as at least 70% of respondents rating an item as ‘Important’ or ‘Very important’.

Additional Files 2 and 7 provide the exact wording of each item.

## Supplementary Table 13: Pre-operative total knee replacement exercise types: Round 2 importance ratings

| **Pre-operative total knee replacement exercise type item^a^** | **All panellists (n = 57)** | | | **Patient panellists (n = 28)** | | | **Professional panellists (n = 29)** | | |
| --- | --- | --- | --- | --- | --- | --- | --- | --- | --- |
|  | **% Important or Very Important rating** | **Median** | **Interquartile range** | **% Important or Very Important rating** | **Median** | **Interquartile range** | **% Important or Very Important rating** | **Median** | **Interquartile range** |
| 3.1 Leg strengthening exercises | 100 | 5 | 0 | 100 | 5 | 0 | 100 | 5 | 0 |
| *3.2 Arm strengthening exercises* | 51 | 4 | 1 | 43 | 3 | 1 | 59 | 4 | 1 |
| 3.3 Leg flexibility exercises | 95 | 5 | 1 | 100 | 5 | 0.75 | 90 | 5 | 1 |
| *3.4 Arm flexibility exercises* | 25 | 3 | 0.5 | 32 | 3 | 1 | 17 | 3 | 0 |
| *3.5 Torso flexibility exercises* | 32 | 3 | 1 | 39 | 3 | 1 | 24 | 3 | 0.5 |
| 3.6 Balance exercises | 95 | 5 | 1 | 96 | 4 | 1 | 93 | 5 | 1 |
| 3.7 Functional movement exercises | 96 | 5 | 1 | 93 | 4 | 1 | 100 | 5 | 0.5 |
| 3.8 Functional technique exercises | 89 | 4 | 1 | 82 | 4 | 0.75 | 97 | 4 | 1 |
| *3.9 Warm-up exercises* | 60 | 4 | 1 | 71 | 4 | 1 | 48 | 3 | 1 |
| *3.10 Cool-down exercises* | 44 | 3 | 1 | 46 | 3 | 1 | 41 | 3 | 1 |
| *3.11 Cardiovascular exercises* | 67 | 4 | 1 | 54 | 4 | 1 | 79 | 4 | 0 |
| *3.12 Core control exercises* | 68 | 4 | 1 | 64 | 4 | 1 | 72 | 4 | 1 |
| 3.13 Walking practice with walking aids | 88 | 5 | 1 | 89 | 5 | 1 | 86 | 5 | 1 |
| 3.14 Training on steps | 91 | 5 | 1 | 100 | 5 | 1 | 83 | 5 | 1 |
| 3.15 Practicing post-operative exercises | 89 | 5 | 1 | 82 | 4 | 1 | 97 | 5 | 1 |
| *3.16 Water-based exercises* | 32 | 3 | 1 | 43 | 3 | 1 | 21 | 3 | 0 |
| *3.17 Exercises in which the foot does not move* | 39 | 3 | 1 | 50 | 3.5 | 1 | 28 | 3 | 2 |

^a^ Items in italics did not reach consensus. Consensus was defined as at least 70% of respondents rating an item as ‘Important’ or ‘Very important’.

## Supplementary Table 14: Pre-operative total knee replacement exercise programme delivery: Round 2 importance ratings

| **Pre-operative total knee replacement exercise programme delivery item^a^** | **All panellists (n = 57)** | | | **Patient panellists (n = 28)** | | | **Professional panellists (n = 29)** | | |
| --- | --- | --- | --- | --- | --- | --- | --- | --- | --- |
|  | **% Important or Very Important rating** | **Median** | **Interquartile range** | **% Important or Very Important rating** | **Median** | **Interquartile range** | **% Important or Very Important rating** | **Median** | **Interquartile range** |
| *4.1.1 Delivered using an individual instruction session* | 53 | 4 | 1 | 68 | 4 | 1 | 38 | 3 | 2 |
| 4.1.2 Delivered using supervised exercise sessions | 79 | 4 | 0 | 82 | 4 | 0 | 76 | 4 | 0.5 |
| 4.1.3 Delivered using unsupervised exercise sessions | 72 | 4 | 1 | 75 | 4 | 0.75 | 69 | 4 | 1 |
| *4.1.4 Delivered using telephone- delivered sessions* | 9 | 3 | 1 | 11 | 3 | 1 | 7 | 3 | 1 |
| 4.1.5 Delivered using a booklet or other written format | 93 | 4 | 1 | 96 | 4 | 0.75 | 90 | 4 | 1 |
| 4.2 Delivered using a combination of more than one format | 96 | 4 | 1 | 96 | 4 | 1 | 97 | 5 | 1 |
| *4.3.1 Take place in the patient’s own home* | 46 | 3 | 3 | 39 | 3 | 2 | 52 | 4 | 2 |
| *4.3.2 Take place in a clinical setting* | 47 | 3 | 1 | 54 | 4 | 1 | 41 | 3 | 1 |
| *4.3.3 Take place in a community setting* | 40 | 3 | 1 | 29 | 3 | 1 | 52 | 4 | 1 |
| *4.4.1 Include high intensity exercises* | 14 | 3 | 1 | 21 | 3 | 1 | 7 | 3 | 1 |
| 4.4.2 Include low to moderate intensity exercises | 91 | 4 | 0 | 86 | 4 | 0 | 97 | 4 | 0 |
| 4.5 Tailored to the patient’s ability | 96 | 5 | 1 | 96 | 5 | 1 | 97 | 5 | 0.5 |
| 4.6 Be progressive | 91 | 4 | 1 | 89 | 4 | 1 | 93 | 4 | 1 |
| *4.7 Each session should last a minimum of 15 minutes* | 63 | 4 | 1 | 68 | 4 | 1 | 59 | 4 | 1 |
| 4.8 Involve a minimum of 2 sessions per week | 79 | 4 | 0.5 | 82 | 4 | 0.75 | 76 | 4 | 1 |
| 4.9 Ideally be performed for a minimum of 6 weeks | 88 | 4 | 1 | 86 | 4 | 1 | 90 | 4 | 0.5 |
| 4.10 Tailored to each patient's needs | 88 | 4 | 1 | 86 | 4 | 1 | 90 | 4 | 1 |
| *4.11 Provide an opportunity for peer support* | 65 | 4 | 1 | 50 | 3.5 | 1 | 79 | 4 | 0.5 |
| 4.12 Include goal setting | 79 | 4 | 1 | 71 | 4 | 1 | 86 | 4 | 1 |

^a^ Items in italics did not reach consensus. Consensus was defined as at least 70% of respondents rating an item as ‘Important’ or ‘Very important’.

Additional Files 2 and 7 provide the exact wording of each item.

## Supplementary Table 15: Other pre-operative total knee replacement treatments: Round 2 importance ratings

| **Other pre-operative total knee replacement treatment item^a^** | **All panellists (n = 57)** | | | **Patient panellists (n = 28)** | | | **Professional panellists (n = 29)** | | |
| --- | --- | --- | --- | --- | --- | --- | --- | --- | --- |
|  | **% Important or Very Important rating** | **Median** | **Interquartile range** | **% Important or Very Important rating** | **Median** | **Interquartile range** | **% Important or Very Important rating** | **Median** | **Interquartile range** |
| *5.1 Patients who have a BMI of 27 kg/m² or over should be referred to a weight management programme* | 67 | 4 | 1 | 57 | 4 | 1 | 76 | 4 | 1 |
| 5.2 Patients who have been formally diagnosed anxiety or depression should be offered CBT-based therapy | 74 | 4 | 1 | 68 | 4 | 1 | 79 | 4 | 1 |
| *5.3 Patients should be offered motivational interviewing* | 37 | 3 | 1 | 21 | 3 | 0 | 52 | 4 | 1 |
| *5.4 Patients should be offered neuromuscular electrical stimulation* | 5 | 3 | 1 | 11 | 3 | 1 | 0 | 2 | 1 |
| *5.5 Patients should be offered electro-acupuncture* | 0 | 2 | 2 | 0 | 2 | 1 | 0 | 2 | 2 |

*BMI* Body Mass Index

*CBT* Cognitive behavioural therapy

^a^ Items in italics did not reach consensus. Consensus was defined as at least 70% of respondents rating an item as ‘Important’ or ‘Very important’.

Additional File 2 provides the exact wording of each item.

# Round 3

## Supplementary Table 16: Pre-operative total knee replacement education topics: Round 3 importance ratings

| **Pre-operative total knee replacement education topic item^a^** | **All panellists (n = 55)** | | | **Patient panellists (n = 26)** | | | **Professional panellists (n = 29)** | | |
| --- | --- | --- | --- | --- | --- | --- | --- | --- | --- |
|  | **% Important or Very Important rating** | **Median** | **Interquartile range** | **% Important or Very Important rating** | **Median** | **Interquartile range** | **% Important or Very Important rating** | **Median** | **Interquartile range** |
| 1.1 Anatomy of the knee joint | 95 | 4 | 0 | 88 | 4 | 0 | 100 | 4 | 0 |
| 1.2 Health conditions that may contribute to needing TKR surgery | 91 | 4 | 1 | 92 | 4 | 0 | 90 | 4 | 1 |
| 1.3 Alternative treatment options to TKR surgery | 87 | 5 | 1 | 85 | 4 | 1 | 90 | 5 | 1 |
| 1.4 Purpose of pre-operative rehabilitation | 98 | 5 | 0 | 96 | 5 | 0 | 100 | 5 | 0 |
| 1.5 Patient involvement in their own management | 98 | 5 | 0 | 96 | 5 | 0 | 100 | 5 | 0 |
| 1.6 Goal setting | 96 | 4 | 1 | 96 | 4 | 1 | 97 | 5 | 1 |
| 1.7 Using heat and cold | 85 | 4 | 0 | 92 | 4 | 1 | 79 | 4 | 0 |
| 1.8 Obtaining and using walking aids and other equipment | 95 | 5 | 1 | 100 | 5 | 0 | 90 | 5 | 1 |
| 1.9 Making home preparations | 100 | 5 | 0 | 100 | 5 | 1 | 100 | 5 | 0 |
| 1.10 Arranging social support | 96 | 5 | 1 | 96 | 5 | 1 | 97 | 5 | 0 |
| 1.11 Arranging transport to and from the hospital | 98 | 5 | 1 | 96 | 5 | 1 | 100 | 5 | 1 |
| 1.12 What to expect during the hospital stay | 100 | 5 | 0 | 100 | 5 | 0 | 100 | 5 | 0 |
| 1.13 What a TKR surgical procedure involves | 93 | 4 | 1 | 92 | 4.5 | 1 | 93 | 4 | 1 |
| 1.14 Risks of TKR surgery and how to minimise them | 100 | 5 | 0 | 100 | 5 | 0 | 100 | 5 | 0 |
| 1.15 Common issues that may occur following TKR surgery which do not need to cause alarm | 100 | 5 | 0 | 100 | 5 | 0.25 | 100 | 5 | 0 |
| 1.16 Pain expectations | 100 | 5 | 0 | 100 | 5 | 0 | 100 | 5 | 0 |
| 1.17 What to expect following discharge | 100 | 5 | 0 | 100 | 5 | 0 | 100 | 5 | 0 |
| 1.18 Recovery expectations | 98 | 5 | 0 | 96 | 5 | 0 | 100 | 5 | 0 |
| 1.19 Pain management | 100 | 5 | 0 | 100 | 5 | 0 | 100 | 5 | 0 |
| 1.20 Rehabilitation following TKR surgery | 100 | 5 | 0 | 100 | 5 | 0 | 100 | 5 | 0 |
| *1.21 Complementary and alternative therapies* | 27 | 3 | 1 | 31 | 3 | 1 | 24 | 3 | 1 |
| 1.22 Returning to daily activities | 98 | 4 | 1 | 96 | 4 | 1 | 100 | 5 | 1 |
| 1.23 Returning to driving and other types of travel | 98 | 4 | 1 | 96 | 4.5 | 1 | 100 | 4 | 1 |
| 1.24 Returning to sports and leisure activities | 96 | 4 | 0 | 92 | 4 | 0 | 100 | 4 | 0 |
| 1.25 Returning to work | 95 | 4 | 1 | 88 | 4 | 1 | 100 | 4 | 1 |
| 1.26 Physical activity | 100 | 5 | 1 | 100 | 4 | 1 | 100 | 5 | 1 |
| 1.27 Weight management | 100 | 5 | 1 | 100 | 4 | 1 | 100 | 5 | 1 |
| 1.28 Stopping smoking | 85 | 5 | 1 | 73 | 4.5 | 2 | 97 | 5 | 1 |
| 1.29 Avoiding alcohol misuse | 87 | 4 | 0 | 81 | 4 | 0.25 | 93 | 4 | 0.5 |
| 1.30 Optimising management of diabetes | 82 | 4 | 1 | 77 | 4 | 1.25 | 86 | 4 | 1 |
| 1.31 Education for other people, such as carers | 91 | 4 | 1 | 85 | 4 | 1 | 97 | 4 | 1 |
| 1.32 Swelling | 100 | 5 | 1 | 100 | 5 | 1 | 100 | 5 | 0 |
| 1.33 Organising help if complications occur | 100 | 5 | 1 | 100 | 5 | 1 | 100 | 5 | 1 |
| 1.34 Returning to a normal walking pattern | 98 | 5 | 1 | 96 | 5 | 1 | 100 | 5 | 1 |
| 1.35 Emotional well-being | 93 | 4 | 1 | 85 | 5 | 1 | 100 | 4 | 0.5 |

*TKR* Total knee replacement

^a^ Item in italics did not reach consensus. Consensus was defined as at least 70% of respondents rating an item as ‘Important’ or ‘Very important’.

## Supplementary Table 17: Pre-operative total knee replacement education delivery: Round 3 importance ratings

| **Pre-operative total knee replacement education delivery item^a^** | **All panellists (n = 55)** | | | **Patient panellists (n = 26)** | | | **Professional panellists (n = 29)** | | |
| --- | --- | --- | --- | --- | --- | --- | --- | --- | --- |
|  | **% Important or Very Important rating** | **Median** | **Interquartile range** | **% Important or Very Important rating** | **Median** | **Interquartile range** | **% Important or Very Important rating** | **Median** | **Interquartile range** |
| 2.1 Informed by a multidisciplinary team | 96 | 4 | 1 | 92 | 4 | 1 | 100 | 5 | 1 |
| 2.2.1 Informed by the orthopaedic surgery team | 95 | 5 | 1 | 92 | 5 | 1 | 97 | 5 | 1 |
| 2.2.2 Informed by the nursing team | 95 | 4 | 1 | 96 | 4 | 1 | 93 | 4 | 1 |
| 2.2.3 Informed by the physiotherapy team | 100 | 5 | 0 | 100 | 5 | 0 | 100 | 5 | 0 |
| 2.2.4 Informed by the occupational therapy team | 85 | 4 | 1 | 77 | 4 | 0.25 | 93 | 4 | 1 |
| *2.2.5 Informed by the social work team* | 31 | 3 | 1 | 23 | 3 | 0.25 | 38 | 3 | 1 |
| 2.3 Informed by patients who have previously had TKR surgery | 73 | 4 | 1 | 73 | 4 | 1 | 72 | 4 | 1 |
| 2.4 Provide examples of other patients’ experiences of TKR surgery | 76 | 4 | 0 | 73 | 4 | 1 | 79 | 4 | 0 |
| 2.5.1 Delivered using face-to-face group sessions | 89 | 4 | 1 | 88 | 4 | 0.25 | 90 | 5 | 1 |
| *2.5.2 Delivered using face-to-face individual sessions* | 56 | 4 | 1 | 54 | 4 | 1 | 59 | 4 | 1 |
| 2.5.3 Delivered using a booklet or other written format | 100 | 4 | 1 | 100 | 4 | 1 | 100 | 5 | 1 |
| *2.5.4 Delivered using a video or DVD* | 64 | 4 | 1 | 62 | 4 | 1 | 66 | 4 | 1 |
| 2.5.5 Delivered using a website or other electronic format | 75 | 4 | 1 | 73 | 4 | 1 | 76 | 4 | 0.5 |
| *2.5.6 Delivered using telephone* | 18 | 3 | 0 | 15 | 3 | 0 | 21 | 3 | 1 |
| *2.5.7 Delivered using a PowerPoint presentation* | 25 | 3 | 1 | 19 | 3 | 0 | 31 | 3 | 1 |
| 2.6 Delivered using a combination of more than one format | 93 | 5 | 1 | 88 | 4 | 1 | 97 | 5 | 0.5 |
| 2.7 Delivered through a combination of information provision and an opportunity to actively take part in tasks | 96 | 4 | 0 | 92 | 4 | 0 | 100 | 4 | 0 |
| 2.8 Provide an opportunity for questions to be addressed | 100 | 5 | 0 | 100 | 5 | 0.25 | 100 | 5 | 0 |
| 2.9 Provide an opportunity for a family member/ friend to be involved | 93 | 4 | 1 | 85 | 4 | 0 | 100 | 5 | 1 |
| 2.10 Tailored to each patient’s needs | 91 | 4 | 0 | 92 | 4 | 0 | 90 | 4 | 0 |
| *2.11 Tailored to the right or left knee* | 2 | 2 | 2 | 4 | 2 | 2 | 0 | 1 | 1 |
| 2.12 Received separately from patients waiting for other types of surgery | 76 | 5 | 1 | 77 | 5 | 1.25 | 76 | 4 | 1.5 |
| 2.13 Delivered within 4 weeks of TKR surgery | 84 | 4 | 1 | 85 | 4 | 1 | 83 | 4 | 0.5 |
| *2.14 Delivered in a hospital setting* | 22 | 3 | 0 | 19 | 3 | 1 | 24 | 3 | 0.5 |
| 2.15 Standardised across the UK | 80 | 4 | 0 | 77 | 4 | 0.25 | 83 | 4 | 0 |

*TKR* Total knee replacement

^a^ Items in italics did not reach consensus. Consensus was defined as at least 70% of respondents rating an item as ‘Important’ or ‘Very important’.

Additional Files 2 and 7 provide the exact wording of each item.

## Supplementary Table 18: Pre-operative total knee replacement exercise types: Round 3 importance ratings

| **Pre-operative total knee replacement exercise type item^a^** | **All panellists (n = 55)** | | | **Patient panellists (n = 26)** | | | **Professional panellists (n = 29)** | | |
| --- | --- | --- | --- | --- | --- | --- | --- | --- | --- |
|  | **% Important or Very Important rating** | **Median** | **Interquartile range** | **% Important or Very Important rating** | **Median** | **Interquartile range** | **% Important or Very Important rating** | **Median** | **Interquartile range** |
| 3.1 Leg strengthening exercises | 100 | 5 | 0 | 100 | 5 | 0 | 100 | 5 | 0 |
| *3.2 Arm strengthening exercises* | 64 | 4 | 1 | 73 | 4 | 1 | 55 | 4 | 1 |
| 3.3 Leg flexibility exercises | 98 | 5 | 0 | 100 | 5 | 0 | 97 | 5 | 1 |
| *3.4 Arm flexibility exercises* | 29 | 3 | 1 | 46 | 3 | 1 | 14 | 3 | 0 |
| *3.5 Torso flexibility exercises* | 27 | 3 | 1 | 42 | 3 | 1 | 14 | 3 | 0 |
| 3.6 Balance exercises | 100 | 5 | 1 | 100 | 4 | 1 | 100 | 5 | 1 |
| 3.7 Functional movement exercises | 95 | 5 | 1 | 92 | 5 | 1 | 97 | 5 | 0.5 |
| 3.8 Functional technique exercises | 91 | 4 | 0 | 85 | 4 | 0 | 97 | 4 | 1 |
| *3.9 Warm-up exercises* | 55 | 4 | 1 | 54 | 4 | 1 | 55 | 4 | 1 |
| *3.10 Cool-down exercises* | 36 | 3 | 1 | 35 | 3 | 1 | 38 | 3 | 1 |
| 3.11 Cardiovascular exercises | 75 | 4 | 1 | 62 | 4 | 1 | 86 | 4 | 0 |
| 3.12 Core control exercises | 76 | 4 | 0 | 69 | 4 | 1 | 83 | 4 | 0 |
| 3.13 Walking practice with walking aids | 91 | 5 | 1 | 85 | 5 | 1 | 97 | 5 | 1 |
| 3.14 Training on steps | 95 | 5 | 1 | 92 | 5 | 1 | 97 | 5 | 1 |
| 3.15 Practicing post-operative exercises | 96 | 5 | 1 | 92 | 4.5 | 1 | 100 | 5 | 1 |
| *3.16 Water-based exercises* | 25 | 3 | 1 | 38 | 3 | 1 | 14 | 3 | 0 |
| *3.17 Exercises in which the foot does not move* | 18 | 3 | 0 | 23 | 3 | 0.25 | 14 | 3 | 0 |

^a^ Items in italics did not reach consensus. Consensus was defined as at least 70% of respondents rating an item as ‘Important’ or ‘Very important’.

## Supplementary Table 19: Pre-operative total knee replacement exercise programme delivery: Round 3 importance ratings

| **Pre-operative total knee replacement exercise programme delivery item^a^** | **All panellists (n = 55)** | | | **Patient panellists (n = 26)** | | | **Professional panellists (n = 29)** | | |
| --- | --- | --- | --- | --- | --- | --- | --- | --- | --- |
|  | **% Important or Very Important rating** | **Median** | **Interquartile range** | **% Important or Very Important rating** | **Median** | **Interquartile range** | **% Important or Very Important rating** | **Median** | **Interquartile range** |
| *4.1.1 Delivered using an individual instruction session* | 44 | 3 | 1 | 54 | 4 | 1 | 34 | 3 | 1 |
| 4.1.2 Delivered using supervised exercise sessions | 89 | 4 | 0 | 88 | 4 | 0 | 90 | 4 | 0 |
| 4.1.3 Delivered using unsupervised exercise sessions | 91 | 4 | 0 | 92 | 4 | 0 | 90 | 4 | 0 |
| *4.1.4 Delivered using telephone- delivered sessions* | 5 | 3 | 1 | 8 | 3 | 1 | 3 | 3 | 1 |
| 4.1.5 Delivered using a booklet or other written format | 93 | 4 | 1 | 88 | 4 | 1 | 97 | 4 | 1 |
| 4.2 Delivered using a combination of more than one format | 91 | 4 | 1 | 85 | 4 | 0 | 97 | 5 | 1 |
| *4.3.1 Take place in the patient’s own home* | 36 | 3 | 2 | 31 | 3 | 2 | 41 | 3 | 2.5 |
| *4.3.2 Take place in a clinical setting* | 47 | 3 | 1 | 46 | 3 | 1 | 48 | 3 | 1 |
| *4.3.3 Take place in a community setting* | 33 | 3 | 1 | 19 | 3 | 0 | 45 | 3 | 1 |
| *4.4.1 Include high intensity exercises* | 22 | 3 | 0 | 23 | 3 | 0.25 | 21 | 3 | 0 |
| 4.4.2 Include low to moderate intensity exercises | 98 | 4 | 0 | 100 | 4 | 0 | 97 | 4 | 0 |
| 4.5 Tailored to the patient’s ability | 96 | 5 | 1 | 96 | 5 | 1 | 97 | 5 | 1 |
| 4.6 Be progressive | 87 | 4 | 1 | 85 | 4 | 1 | 90 | 4 | 1 |
| 4.7 Each session should last a minimum of 15 minutes | 80 | 4 | 0 | 85 | 4 | 0 | 76 | 4 | 1 |
| 4.8 Involve a minimum of 2 sessions per week | 84 | 4 | 0 | 88 | 4 | 0 | 79 | 4 | 0 |
| 4.9 Ideally be performed for a minimum of 6 weeks | 89 | 4 | 1 | 85 | 4 | 1 | 93 | 4 | 0 |
| 4.10 Tailored to each patient's needs | 93 | 4 | 1 | 96 | 4 | 1 | 90 | 5 | 1 |
| 4.11 Provide an opportunity for peer support | 75 | 4 | 1 | 65 | 4 | 1 | 83 | 4 | 0 |
| 4.12 Include goal setting | 87 | 4 | 0 | 85 | 4 | 0 | 90 | 4 | 1 |

^a^ Items in italics did not reach consensus. Consensus was defined as at least 70% of respondents rating an item as ‘Important’ or ‘Very important’.

Additional Files 2 and 7 provide the exact wording of each item.

## Supplementary Table 20: Other pre-operative total knee replacement treatments: Round 3 importance ratings

| **Other pre-operative total knee replacement treatment item^a^** | **All panellists (n = 55)** | | | **Patient panellists (n = 26)** | | | **Professional panellists (n = 29)** | | |
| --- | --- | --- | --- | --- | --- | --- | --- | --- | --- |
|  | **% Important or Very Important rating** | **Median** | **Interquartile range** | **% Important or Very Important rating** | **Median** | **Interquartile range** | **% Important or Very Important rating** | **Median** | **Interquartile range** |
| 5.1 Patients who have a BMI of 27 kg/m² or over should be referred to a weight management programme | 73 | 4 | 1 | 62 | 4 | 1 | 83 | 4 | 0 |
| 5.2 Patients who have been formally diagnosed anxiety or depression should be offered CBT-based therapy | 78 | 4 | 0 | 69 | 4 | 1 | 86 | 4 | 0 |
| *5.3 Patients should be offered motivational interviewing* | 33 | 3 | 1 | 23 | 3 | 0.25 | 41 | 3 | 1 |
| *5.4 Patients should be offered neuromuscular electrical stimulation* | 4 | 3 | 1 | 4 | 3 | 1 | 3 | 2 | 1 |
| *5.5 Patients should be offered electro-acupuncture* | 0 | 2 | 2 | 0 | 2 | 1 | 0 | 2 | 1 |

*BMI* Body Mass Index

*CBT* Cognitive behavioural therapy

^a^ Items in italics did not reach consensus. Consensus was defined as at least 70% of respondents rating an item as ‘Important’ or ‘Very important’.

Additional File 2 provides the exact wording of each item.
