# Supplementary material for: Consensus on pre-operative total knee replacement education and prehabilitation recommendations: a UK-based modified Delphi study
Source: BMC Musculoskelet Disord. 2021 Apr 14;22:352. doi: 10.1186/s12891-021-04160-5 (PMC8044503; doi:10.1186/s12891-021-04160-5)
Supplement: Supplementary file 10 — Additional file 10: Prioritised list of recommendations. Prioritised list of recommendations developed from the Round 3 results (Supplementary Tables 21–25). [file 12891_2021_4160_MOESM10_ESM.docx]

**Consensus on pre-operative total knee replacement education and prehabilitation recommendations: A UK-based modified Delphi study**

**Additional File 10: Prioritised list of recommendations**

| **Very important recommendations** |
| --- |
| Very important recommendations were rated as ‘Very important’ by at least 70% of panel members during the final Delphi study round.  The number on the right indicates the percentage of panel members who selected ‘Very important’ for that recommendation during the final Delphi study round. |
| **Important recommendations** |
| Important recommendations were rated as ‘Important’ or ‘Very important’ by at least 70% of panel members during the final Delphi study round.  The number on the right indicates the percentage of panel members who selected ‘Important’ or ‘Very important’ for that recommendation during the final Delphi study round. |
| **Excluded recommendations** |
| Excluded recommendations were rated as ‘Important’ or ‘Very important’ by less than 70% of panel members during the final Delphi study round.  The number on the right indicates the percentage of panel members who selected ‘Important’ or ‘Very important’ for that recommendation during the final Delphi study round. |

# Supplementary Table 21: Pre-operative total knee replacement (TKR) education topics

| **Very important recommendation** | |
| --- | --- |
| At a minimum, pre-operative TKR education should include the following topics: | |
| - Rehabilitation following TKR surgery | 98 |
| - Patient involvement in their own management | 96 |
| - Pain management | 96 |
| - Risks of TKR surgery and how to minimise them | 95 |
| - Recovery expectations | 93 |
| - Pain expectations | 91 |
| - What to expect following discharge | 91 |
| - Purpose of pre-operative rehabilitation | 87 |
| - What to expect during the hospital stay | 85 |
| - Common issues that may occur following TKR surgery which do not need to cause alarm | 85 |
| - Making home preparations | 82 |
| - Arranging social support | 75 |
| **Important recommendation** | |
| At a minimum, pre-operative TKR education should include the following topics: | |
| - Weight management | 100 |
| - Physical activity | 100 |
| - Swelling | 100 |
| - Organising help if complications occur | 100 |
| - Arranging transport to and from the hospital | 98 |
| - Returning to driving and other types of travel | 98 |
| - Returning to daily activities | 98 |
| - Returning to a normal walking pattern | 98 |
| - Goal setting | 96 |
| - Returning to sports and leisure activities | 96 |
| - Anatomy of the knee joint | 95 |
| - Obtaining and using walking aids and other equipment | 95 |
| - Returning to work | 95 |
| - What a TKR surgical procedure involves | 93 |
| - Emotional well-being | 93 |
| - Health conditions that may contribute to needing TKR surgery | 91 |
| - Education for other people, such as carers | 91 |
| - Avoiding alcohol misuse | 87 |
| - Alternative treatment options to TKR surgery | 87 |
| - Using heat and cold | 85 |
| - Stopping smoking | 85 |
| - Optimising management of diabetes | 82 |
| **Excluded recommendation** | |
| At a minimum, pre-operative TKR education should include the following topic: | |
| - Complementary and alternative therapies | 27 |

# Supplementary Table 22: Pre-operative total knee replacement (TKR) education delivery

| **Very important recommendations** | |
| --- | --- |
| At a minimum, pre-operative TKR education should be informed by members of the physiotherapy team | 84 |
| Pre-operative TKR education should provide an opportunity for the patient’s questions to be addressed | 84 |
| **Important recommendations** | |
| At a minimum, pre-operative TKR education should be delivered using a booklet or other written format | 100 |
| Pre-operative TKR education should be informed by a multi-disciplinary team rather than members of a single profession | 96 |
| Pre-operative TKR education should be delivered through a combination of providing the patient with information and giving them an opportunity to actively take part in tasks | 96 |
| At a minimum, pre-operative TKR education should be informed by members of the orthopaedic surgery team | 95 |
| At a minimum, pre-operative TKR education should be informed by members of the nursing team | 95 |
| Pre-operative TKR education should be delivered using a combination of more than one format | 93 |
| Pre-operative TKR education should provide an opportunity for a family member or friend of the patient to be involved | 93 |
| Pre-operative TKR education should be tailored according to each patient’s individual needs | 91 |
| At a minimum, pre-operative TKR education should be delivered using face-to-face group sessions | 89 |
| At a minimum, pre-operative TKR education should be informed by members of the occupational therapy team | 85 |
| At a minimum, at least some pre-operative TKR education should be delivered within 4 weeks of the patient’s TKR surgery | 84 |
| Pre-operative TKR education should be standardised across the United Kingdom | 80 |
| At least some pre-operative TKR education should be delivered by providing examples of other patients’ experiences of TKR surgery | 76 |
| Patients waiting for TKR surgery should receive pre-operative education separately from patients waiting for other types of surgery, such as total hip replacement surgery | 76 |
| At a minimum, pre-operative TKR education should be delivered using a website or other electronic format | 75 |
| Pre-operative TKR education should be informed by patients who have previously had TKR surgery | 73 |
| **Excluded recommendations** | |
| At a minimum, pre-operative TKR education should be delivered using a video or DVD | 64 |
| At a minimum, pre-operative TKR education should be delivered using face-to-face individual sessions | 56 |
| At a minimum, pre-operative TKR education should be informed by members of the social work team | 31 |
| At a minimum, pre-operative TKR education should be delivered using a PowerPoint presentation | 25 |
| At a minimum, at least some pre-operative TKR education should be delivered in a hospital setting, such as a ward | 22 |
| At a minimum, pre-operative TKR education should be delivered using telephone | 18 |
| Pre-operative TKR education should be tailored according to whether the patient is having their right or their left knee replaced | 2 |

# Supplementary Table 23: Pre-operative total knee replacement (TKR) exercise types

| **Very important recommendation** | |
| --- | --- |
| At a minimum, a pre-operative TKR exercise programme should include the following types of exercise: | |
| - Leg strengthening | 89 |
| - Leg flexibility | 78 |
| **Important recommendation** | |
| At a minimum, a pre-operative TKR exercise programme should include the following types of exercise: | |
| - Balance | 100 |
| - Practicing post-operative exercises | 96 |
| - Functional movement | 95 |
| - Training on steps | 95 |
| - Walking practice with walking aids | 91 |
| - Functional technique | 91 |
| - Core control | 76 |
| - Cardiovascular | 75 |
| **Excluded recommendation** | |
| At a minimum, a pre-operative TKR exercise programme should include the following types of exercise: | |
| - Arm strengthening | 64 |
| - Warm-up | 55 |
| - Cool-down | 36 |
| - Arm flexibility | 29 |
| - Torso flexibility | 27 |
| - Water-based exercises | 25 |
| - Exercises in which the foot does not move | 18 |

# Supplementary Table 24: Pre-operative total knee replacement (TKR) exercise programme delivery

| **Important recommendations** | |
| --- | --- |
| At a minimum, a pre-operative TKR exercise programme should include exercises which are low to moderate intensity | 98 |
| A pre-operative TKR exercise programme should be tailored according to the patient’s ability | 96 |
| At a minimum, a pre-operative TKR exercise programme should be delivered using a booklet or other written format | 93 |
| A pre-operative TKR exercise programme should be tailored according to each patient’s individual needs | 93 |
| At a minimum, a pre-operative TKR exercise programme should be delivered using unsupervised exercise sessions | 91 |
| A pre-operative TKR exercise programme should be delivered using a combination of more than one format | 91 |
| A pre-operative TKR exercise programme should ideally be performed for a minimum of six weeks | 89 |
| At a minimum, a pre-operative TKR exercise programme should be delivered using supervised exercise sessions | 89 |
| A pre-operative TKR exercise programme should be progressive | 87 |
| A pre-operative TKR exercise programme should include goal setting | 87 |
| A pre-operative TKR exercise programme should involve a minimum of two exercise sessions per week | 84 |
| Each session in a pre-operative TKR exercise programme should last a minimum of fifteen minutes | 80 |
| A pre-operative TKR exercise programme should provide an opportunity for peer support | 75 |
| **Excluded recommendations** | |
| At a minimum, at least some pre-operative TKR exercise sessions should take place in a clinical setting, such as a hospital or GP practice | 47 |
| At a minimum, a pre-operative TKR exercise programme should be delivered using an individual instruction session | 44 |
| At a minimum, at least some pre-operative TKR exercise sessions should take place in the patient’s own home | 36 |
| At a minimum, at least some pre-operative TKR exercise sessions should take place in a community setting, such as a sports centre | 33 |
| At a minimum, a pre-operative TKR exercise programme should include exercises which are high intensity | 22 |
| At a minimum, a pre-operative TKR exercise programme should be delivered using telephone delivered sessions | 5 |

# Supplementary Table 25: Other pre-operative total knee replacement (TKR) treatments

| **Important recommendations** | |
| --- | --- |
| Patients waiting for TKR surgery who have been formally diagnosed anxiety or depression should be offered cognitive behavioural therapy (CBT)-based therapy | 78 |
| Patients waiting for TKR surgery who have a BMI of 27 kg/m² or over should be referred to a weight management programme | 73 |
| **Excluded recommendations** | |
| Patients waiting for TKR surgery should be offered motivational interviewing | 33 |
| Patients waiting for TKR surgery should be offered Neuromuscular Electrical Stimulation (NMES) | 4 |
| Patients waiting for TKR surgery should be offered electroacupuncture | 0 |
